# DNA methylation signature of smoking in lung cancer is enriched for exposure signatures in newborn and adult blood

**DOI:** 10.1038/s41598-019-40963-2

**Published:** 2019-03-14

**Authors:** K. M. Bakulski, J. Dou, N. Lin, S. J. London, J. A. Colacino

**Affiliations:** 1 Department of Epidemiology, School of Public Health, University of Michigan, Ann Arbor, Michigan USA; 2 National Institute of Environmental Health Sciences, National Institutes of Health, Department of Health and Human Services, Research Triangle Park, North Carolina, USA; 3 Department of Environmental Health Sciences, School of Public Health, University of Michigan, Ann Arbor, Michigan USA; 4 Department of Nutritional Sciences, School of Public Health, University of Michigan, Ann Arbor, Michigan USA; 5 Center for Computational Medicine and Bioinformatics, University of Michigan, Ann Arbor, Michigan USA

## Abstract

Smoking impacts DNA methylation genome-wide in blood of newborns from maternal smoking during pregnancy and adults from personal smoking. We compared smoking-related DNA methylation in lung adenocarcinoma (61 never smokers, 91 current smokers, and 238 former smokers) quantified with the Illumina450k BeadArray in The Cancer Genome Atlas with published large consortium meta-analyses of newborn and adult blood. We assessed whether CpG sites related to smoking in blood from newborns and adults were enriched in the lung adenocarcinoma methylation signal. Testing CpGs differentially methylated by smoke exposure, we identified 296 in lung adenocarcinoma meeting a *P* < 10^−4^ cutoff, while previous meta-analyses identified 3,042 in newborn blood, and 8,898 in adult blood meeting the same *P* < 10^−4^ cutoff. Lung signals were highly enriched for those seen in newborn (24 overlapping CpGs, P_*enrichment*_ = 1.2 × 10^−18^) and adult blood (66 overlapping CpGs, *P*_*enrichment*_ = 1.2 × 10^−48^). The 105 genes annotated to CpGs differentially methylated in lung tumors, but not blood, were enriched for RNA processing ontologies. Some epigenetic alterations associated with cigarette smoke exposure are tissue specific, but others are common across tissues. These findings support the value of blood-based methylation biomarkers for assessing exposure effects in target tissues.

## Introduction

Approximately one quarter of cancer deaths are attributable to tobacco use^1^. The lung is the primary tissue affected by tobacco smoke and tobacco accounts for 87% of deaths due to lung cancer^1^. Specifically, lung adenocarcinoma, a type of non-small cell lung cancer, is the leading cause of cancer deaths globally. Epigenetic modifications, including DNA methylation, are widely detected in cancers including lung adenocarcinoma and may play a role in pathogenesis^2^.

Exposure to cigarette smoke is associated with altered DNA methylation at many locations throughout the genome. A recent epigenome-wide meta-analysis of blood DNA methylation using the Illumina450K Beadchip in 6,685 newborns from 13 studies in the Pregnancy and Child Epigenetics (PACE) consortium identified over 6,000 CpG sites differentially methylated in relation to maternal smoking during pregnancy^3^. These differential blood DNA methylation patterns were subsequently shown to be a reliable biomarker of maternal smoking exposure in newborns^4^. In adults, personal smoking was related to widespread differential blood methylation in a meta-analysis of 16 cohorts (n = 15,907) in the Cohorts for Heart and Aging Research in Genetic Epidemiology (CHARGE) consortium^5^. Exposure to cigarette smoke is associated with reproducible and specific DNA methylation changes in newborn and adult blood.

While blood is readily available in large scale population studies, the target tissues for the diseases of interest are not. A few studies have compared blood smoking candidate gene DNA methylation associations to lung, the primary organ exposed to smoke and the major target of smoking related carcinogenesis. In a subset of the European Prospective Investigation into Cancer and Nutrition (n = 374), two candidate CpGs in the Aryl-Hydrocarbon Receptor Repressor (*AHRR*) gene were differentially methylated in relation to smoking in blood as well as differentially methylated in human lung tissue, with the same direction of effect^6^. An epigenome-wide association study of tobacco smoke exposure in lung tissue found eight CpG sites with reduced methylation in smokers, five of which had been previously identified in studies examining blood DNA methylation in relation to smoking^7^. However, the smoking signatures from newborn or adult blood have not been systematically compared with signatures in a highly relevant target tissue for smoking related health effects, the lung.

Using published meta-analysis results from the PACE and CHARGE consortia, we compared smoking-related DNA methylation signatures detectable in blood to those in a well-characterized collection of lung adenocarcinoma cases from The Cancer Genome Atlas (TCGA). We sought to test whether the blood-based DNA methylation smoking signals at birth, from *in utero* exposure, and in adulthood, from personal exposure, are reflective of smoking-related methylation in lung tumor tissue.

## Methods

### Ethical approval and consent to participate

This study was conducted using publicly available data for secondary data analysis. The lung cancer data were collected by The Cancer Genome Atlas (TCGA). The National Cancer Institute (NCI) and National Human Genome Research Institute (NHGRI) worked with physicians who collected tissue for TCGA to gain ethical approval with local Institutional Review Boards and informed consent from participants. The newborn blood findings were produced by the PACE consortium and the adult blood findings were generated by the CHARGE consortium where written informed consent was obtained for all participants and ethical approvals were obtained by the participating studies.

### Lung adenocarcinoma study sample

In The Cancer Genome Atlas a total of 507 samples were obtained at surgery from individuals with lung adenocarcinoma^2^. Smoking status was assessed by questionnaire (never, current, former > 15 years, former ≤ 15 years). DNA was extracted and bisulfite converted as previously described^2^. DNA methylation was measured using the Illumina Infinium HumanMethylation450 BeadChip Kit (450 k)^8^, a validated tool for quantifying genome-scale DNA methylation^9^. Lung adenocarcinoma samples were interspersed across 20 plates with samples from other tissues.

### Lung DNA methylation data preprocessing

Raw methylation image files were downloaded from the Genomic Data Commons (GDC). We calculated and analyzed methylated (M) and unmethylated (U) intensities for low-quality samples (M < 11, U < 11) (n = 37). Samples were removed if more than 1% of probes did not meet a detection *P*-value of 0.01 (n = 43). Probes with noted cross-reactivity^10^ (n = 29,233) and probes with low detection (*P* < 0.01 in >10% of samples, n = 3,503) were removed. Some individuals had multiple samples with methylation measures (n = 32). For these individuals, we selected the sample with the smallest proportion of probes failing detection *P*-value. We used normal-exponential using out-of-band probe (noob) background correction^11^, and resulting beta values of methylation were utilized for subsequent analysis. We flagged “gap probes” that have clustered methylation distributions, likely SNP-associated probes influenced by underlying genetic variation, using the gap hunter function in minfi with 5% as the distance threshold to define gaps and 1% as the outlier group cutoff^12^. A summary flowchart of sample and probe exclusion can be found in Supplementary Fig. 1.

### Relation of lung DNA methylation to smoking

We tested for an association between DNA methylation and categorical smoking status, either current or former (recent ≤ 15 year quitters and longer >15 year quitters as separate groups) with reference to never smoking using multivariable linear regression in limma^13^, with empirical Bayesian standard error adjustment^14^. We adjusted for sex, age, cancer stage, plate, and the first ten principal components of ancestry^15^. In a sensitivity model, we used surrogate variable adjustment to account for unmeasured confounding and potential batch effects^16^. We generated plots of observed versus expected *P*-values and calculated lambda genomic inflation^17^. We examined if methylation findings in lung cancer overlapped with SNPs found to be associated with lung cancer in genome-wide association studies by comparing to results compiled in the GWAS Catalog, a curated database of published SNP-trait associations^18^. We downloaded a list of 827 SNPs with lung carcinoma as the associated trait (accessed 27 November 2018) and compared the 845 genes mapped to those SNPs to genes mapped to CpG sites significantly associated with smoking.

### Smoking signatures in newborn and adult blood

Published results from previous, large epigenome-wide association meta-analyses were used to define smoking related blood DNA methylation signatures. In the PACE consortium, maternal sustained smoking (not including women who quit early in pregnancy) was associated with newborn blood 450 k DNA methylation in 13 cohorts^3^. Meta-analysis results for the 6,073 CpGs significantly related to maternal smoking (False Discovery Rate (FDR) q value < 0.05, corresponding to *P* < 6.5 × 10^−4^) were available. Of these 6,073, data were available for 5,936 CpGs in the analysis of lung adenocarcinoma. In the CHARGE consortium, current smoking status was associated with adult blood 450 k DNA methylation in 16 cohorts^5^. Results from the 18,760 probes significantly related to smoking (FDR q value < 0.05, corresponding to *P* < 1.9 × 10^−3^) in CHARGE were available. Of these 18,760 smoking associated probes, data were also available for 18,126 probes in the lung adenocarcinoma analyses. We used site-specific smoking effect estimates, standard errors, and *P*-values from each of the two studies.

### Enrichment testing

Our primary analysis of DNA methylation in lung adenocarcinoma compared current smokers to never smokers. We first tested all pairwise Pearson correlations between effect estimates or *P*-values for the newborn blood sustained smoke exposure, adult current smoking, and lung current smoking results. We examined enrichment of the blood smoking signatures in the lung results by looking at the overlap of CpG sites that met significance thresholds. In our primary analysis, the *P*-value cutoff for probes used was 10^−4^. Fisher’s exact tests were used to determine significance of the overlap between lung and blood associated CpGs. Given our modest sample size in lung adenocarcinoma tissue (n = 390), a liberal value of *P* < 10^−4^ was used to include a sufficient number of sites for analysis across tissues. As a sensitivity test, we also repeated enrichment analysis using FDR < 0.05 cutoffs across tissues. FDR cutoffs, however, corresponded to significance levels that varied by large magnitudes across the tissues: 6.5 × 10^−4^, 1.9 × 10^−3^ and 6.9 × 10^−6^ in newborn blood, adult blood, and lung adenocarcinoma, respectively. In addition, we varied the *P*-value cutoff for inclusion of probes in the lung smoking signature from 1.0 to 10^−10^ in order to evaluate sensitivity to our choice of threshold. We used the Illumina 450 k annotation file to compare enriched sites by genomic region (CpG island, shore, shelf, or open sea).

### Gene ontology analysis

We examined the annotated genes of CpG sites that were uniquely differentially methylated in lung adenocarcinoma of current smokers for enriched gene ontologies. We further tested for enriched ontologies from genes annotated to smoking-associated CpG sites that overlapped between lung adenocarcinoma and either newborn or adult blood. A *P*-value cutoff of 10^−4^ was used to determine CpG sites for inclusion in gene ontology analysis using the missMethyl package^19^. We compared ontology findings from the two sets of analyses performed: one considering genes with differentially methylated sites in lung only, and a second that considered genes with differentially methylated sites in both lung adenocarcinoma and blood. Restricting to biological pathways with more than five genes, REVIGO was used to remove redundant gene ontologies^20^. To evaluate whether these pathway enrichment results are reflections of pathways dominating in the overall lung current smoker results, we performed pathway analysis with permutations of randomly selected subsets of the lung smoking CpGs, ignoring blood overlap or lung uniqueness.

### Secondary analyses

We tested for association in lung adenocarcinoma among former smokers at two different durations of time since quitting smoking (≤15 years and >15 years) relative to never smokers. We repeated the correlation and enrichment testing as specified above. We performed 10,000 permutations where we randomly reordered *P*-values to new CpGs and tested for enrichment. Enrichment for blood signals was also done stratified by cancer stage to investigate possible changes by cancer progression.

We repeated analysis for enrichment of blood smoking signals in TCGA urothelial bladder carcinoma samples. Sample and probe exclusion for these samples are also summarized in Supplementary Fig. 1. Models testing for single site associations with smoking status in bladder cancer patients were adjusted for the same covariates used in lung models. We also examined the overlap between top smoking-associated CpGs in lung to those in bladder.

## Results

### Lung adenocarcinoma study sample descriptive statistics

After preprocessing, DNA methylation data were available on lung adenocarcinomas from 423 individuals in the TCGA. We analyzed the 390 participants with data on self-reported smoking status and covariates of interest (Table 1). The study sample was 54.9% female, 82.3% White, and 55.4% had cancer stage 1. Participants were a mean age of 65.3 at surgery. Smoking status differed by sex and age (Table 2).

**Table 1:** Univariate descriptive statistics of The Cancer Genome Atlas lung adenocarcinoma tissue study sample.

Covariate	Analytic sample (n = 390)
Smoking status *N(%)*	
Never	61 (15.6)
Current	91 (23.3)
Former (≤15 years)	135 (34.6)
Former (>15 years)	103 (26.4)
Sex *N(%)*	
Male	176 (45.1)
Female	214 (54.9)
Age *Mean(IQR)*	65.3 (14)
Smoking pack years *Mean(IQR)*	42.2 (27.5)
Missing *N(%)*	113 (29.0)
Race *N(%)*	
White	321 (82.3)
Black	35 (9.0)
Asian	6 (1.5)
Unknown	28 (7.2)
Cancer stage *N(%)*	
I	216 (55.4)
II	93 (23.8)
III	62 (15.9)
IV	19 (4.9)

We report mean(IQR) for continuous covariates and frequency (percent) for categorical covariates.

**Table 2:** Bivariate descriptive statistics in the lung adenocarcinoma study (N = 390).

Covariate	Never smokers N = 61	Current smokers N = 91	Former ≤ 15 years N = 135	Former > 15 years N = 103	P-value
Sex N(%)					0.0027
Male	17 (27.9)	52 (57.1)	56 (41.5)	51 (49.5)	
Age Mean(IQR)	65.6 (14)	61.4 (13)	63.9 (12)	70.5 (10)	3.27 × 10^−10^
Smoking pack years Mean(IQR)	—	52.1 (28.0)	42.8 (26.5)	32.5 (24.25)	4.02 × 10^−5^
Missing (%)	—	16 (17.6)	17 (12.6)	19 (18.4)	
Race N(%)					0.66
White	52 (85.2)	73 (80.2)	108 (80.0)	88 (85.4)	
Black	3 (4.9)	11 (12.1)	13 (9.6)	8 (7.8)	
Asian	2 (3.3)	2 (2.2)	2 (1.5)	0 (0.0)	
Unknown	4 (6.6)	5 (5.5)	12 (8.9)	7 (6.8)	
Cancer stage N(%)					0.58
I	30 (49.2)	49 (53.8)	75 (55.6)	62 (60.2)	
II	17 (27.9)	27 (29.7)	27 (20.0)	22 (21.4)	
III	9 (14.8)	11 (12.1)	26 (19.3)	16 (15.5)	
IV	5 (8.2)	4 (4.4)	7 (5.2)	3 (2.9)	

We report mean (IQR) for continuous covariates and frequency (percent) for categorical covariates and test for differences among the smoking categories, using Fisher’s test for categorical variables and ANOVA for continuous variables.

### Smoking status and DNA methylation in lung adenocarcinoma samples

For the primary model of current smoking versus never smoking in relation to DNA methylation, we did not observe major inflation (lambda = 1.13) (Supplementary Fig. 2). The lambda value was slightly reduced by adding 10 surrogate variables to the primary model (current smoker lambda = 1.08). In our primary model, comparing to never smokers, we observed lambda values of 1.05 for recent former smokers and 0.99 for longer-term former smokers (Supplementary Fig. 3). Beta coefficients in the surrogate variable model and the primary model had Pearson correlation of 0.90 (Supplementary Fig. 4). Results across all probes can be found in Supplementary Dataset 1.

In lung adenocarcinoma samples, there were 14 CpGs associated with current smoking status at a Bonferroni adjusted genome-wide significance level (*P* < 10^−7^) (Table 3), 66 CpGs associated with current smoking stats at FDR significance (Supplementary Table 1), and 34,795 CpGs associated at a nominal level (*P* < 0.05) (Supplementary Fig. 5). Second, we tested lung adenocarcinoma samples for differences between recent former smokers (≤15 years) and never smokers. There were 12 CpGs associated with recent former smoking status at a genome-wide significance level (*P* < 10^−7^), 19 sites associated with recent former smoking at FDR < 0.05 and 29,480 CpGs associated at a nominal significance level (*P* < 0.05). Last, we tested for differences between longer term former smokers (>15 years) and never smokers. In former smokers quitting >15 years ago, there were three genome-wide (*P* < 10^−7^) significant sites, 14 FDR significant sites, and 24,620 CpGs were associated with longer former smoking at a nominal level (*P* < 0.05). Comparing genes mapped to FDR significant smoking-related CpG sites and genes implicated in lung cancer risk from published SNP findings in genome-wide association studies compiled in the GWAS Catalog, two genes overlapped: *MYO1G* and *C6orf48*.

**Table 3:** Genome-wide significant CpG sites (*P* < 10^−7^) for smoking versus never smoking in lung adenocarcinoma samples.

CpG	Chr	Position	Annotated Genes	Estimated Difference in % Methylation	Std. Error	P-value	Average % Meth.	Gap Probe
**Current Smoking**								
cg12086028	chr6	33241026	VPS52; RPS18	−15.12	2.37	5.53E-10	31.5	N
cg27033919	chr11	62622173	SNORD30; SNORD29; SLC3A2; SNORD31; SNORD28; SNHG1	−13.19	2.16	2.79E-09	13.4	Y
cg18806997	chr1	45242078	SNORD46; RPS8; SNORD38A	−7.2	1.19	3.34E-09	9.6	N
cg22132788	chr7	45002486	MYO1G	13.31	2.24	7.16E-09	76.3	N
cg16290996	chr1	173835989	GAS5; SNORD78; SNORD76; SNORD77; SNORD44; SNORD79	−12.64	2.13	7.26E-09	21.8	N
cg13985198	chr1	45242073	SNORD46; RPS8; SNORD38A	−8.22	1.39	7.63E-09	6	Y
cg02607319	chr7	45002112		12.95	2.23	1.36E-08	69.2	N
cg07362537	chr6	33240820	VPS52; RPS18	−13.18	2.35	3.96E-08	21.2	Y
cg02905828	chr11	62622234	SNORD30; SNORD29; SLC3A2; SNORD31; SNORD28; SNHG1	−13.25	2.37	4.36E-08	16.5	N
cg11374355	chr19	39925660	RPS16	−9.26	1.66	5.27E-08	42.1	N
cg12973930	chr19	57049695	ZFP28	−10.66	1.92	5.73E-08	11.5	N
cg19089201	chr7	45002287	MYO1G	11.88	2.16	7.42E-08	68	N
cg09345320	chr11	62622179	SNORD30; SNORD29; SLC3A2; SNORD31; SNORD28; SNHG1	−13.27	2.43	8.39E-08	13	Y
cg05840553	chr9	130212635	RPL12; LRSAM1; LRSAM1	−6.28	1.15	8.52E-08	6.8	Y
**Former Smoking (**≤**15 Years)**								
cg22132788	chr7	45002486	MYO1G	15.03	2.05	1.57E-12	76.3	N
cg02607319	chr7	45002112		14.79	2.03	2.31E-12	69.2	N
cg19089201	chr7	45002287	MYO1G	13.17	1.97	9.73E-11	68	N
cg04180046	chr7	45002736	MYO1G	12.01	1.91	1.03E-09	57.8	N
cg23160522	chr15	75015787	CYP1A1	−8.68	1.44	4.34E-09	57.9	N
cg18806997	chr1	45242078	SNORD46; RPS8; SNORD38A	−6.12	1.08	3.34E-08	9.6	N
cg13985198	chr1	45242073	SNORD46; RPS8; SNORD38A	−7.14	1.27	3.69E-08	6	Y
cg18092474	chr15	75019302	CYP1A1	−12.32	2.21	4.65E-08	43.6	N
cg01410359	chr2	38302230	CYP1B1	−10.14	1.82	4.66E-08	33.5	N
cg03224163	chr7	139420300	HIPK2	−3.89	0.7	6.01E-08	83.1	Y
cg09799983	chr2	38301756	CYP1B1	−23.47	4.25	6.59E-08	39.3	N
cg12803068	chr7	45002919	MYO1G	9.37	1.72	9.84E-08	73.4	N
**Former Smoking (**>**15 Years)**								
cg25157280	chr3	48700498	CELSR3	−8.97	1.57	2.27E-08	5.9	N
cg22132788	chr7	45002486	MYO1G	11.88	2.17	8.43E-08	76.3	N
cg09736162	chr3	48700443	CELSR3	−9.35	1.71	9.34E-08	9.1	N

### Correlation of CpGs differentially methylated in related to smoking in adult blood, newborn blood, and lung adenocarcinoma

We next sought to compare the overall pattern of smoking related DNA methylation pairwise across the three tissues: adult blood, newborn blood, and lung adenocarcinoma. First, there were 1,376 CpG sites that were FDR significant (FDR < 0.05) in both the adult blood and the newborn blood meta-analyses. At these sites, adult blood and newborn blood smoking effect estimates were highly correlated (Pearson r = 0.57, *P* < 1.1 × 10^−16^, n = 1,376). Across the 5,936 FDR significant CpG sites in newborn blood that were present in the adenocarcinoma dataset at any level of significance, the effect estimates describing the relationship between current versus never smoking in lung adenocarcinoma and estimates in newborn blood for maternal smoking in pregnancy were only weakly, albeit significantly, correlated (Pearson r = 0.04, *P* = 0.0034). For FDR significant (FDR < 0.05) probes in adult blood, there was slightly stronger correlation between effect estimates for personal smoking in adult lung and blood (Pearson r = 0.13, *P* < 1.1 × 10^−16^, n = 18,126) (Supplementary Fig. 6). The −log10 *P* value ranks for differential methylation findings in lung and blood had a low level of correlation (Spearman rho = 0.03 for lung and adult blood, rho = 0.05 for lung and newborn blood, both *P* < 1.1 × 10^−16^).

### Lung adenocarcinoma smoking DNA methylation signature is enriched for adult blood smoking DNA methylation signature

**Figure 1:**
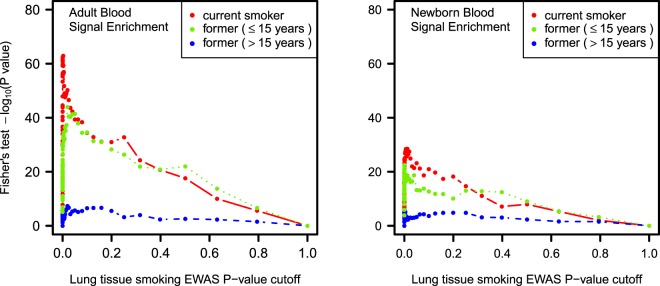
Enrichment for blood smoking associated DNA methylation signal in lung adenocarcinoma. Enrichment was evaluated by testing the overlap of smoking associated sites in lung adenocarcinoma of current smokers (red), recent former smokers (green), and longer former smokers (blue) that met *P* thresholds with smoking associated sites in blood. The threshold in adult and newborn blood meta-analyses was *P* < 10^−4^. In lung, a range of *P* thresholds from 0 to 1 were applied and plotted against Fisher’s test *P-*values.

**Figure 2:**
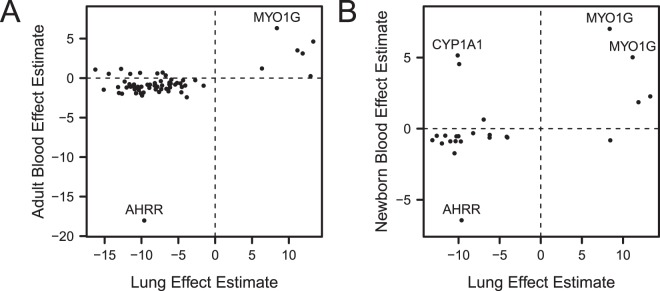
Effect estimates of CpG sites with *P* < 10^−4^. (**A**) CpG sites that are both associated with current smoking in adult blood and current smoking status in lung adenocarcinoma (*P* < 10^−4^) relative to never smokers. (**B**) Effect estimates of CpG sites that are both associated with maternal smoking in newborn blood and current smoking status in lung adenocarcinoma (*P* < 10^−4^) relative to never smokers.

We compared the individual CpG sites associated with smoking in lung adenocarcinoma and adult blood. A total of 8,898 CpG sites associated with current smoking in adult blood with *P* < 10^−4^ were represented in the lung adenocarcinoma dataset. We tested the differentially methylated CpGs in lung for enrichment of those signals in adult blood using variable cutoffs in lung adenocarcinoma results (Fig. 1), and here we report results using the same *P* < 10^−4^ threshold in lung. Among CpG sites distinguishing current smokers versus never smokers in lung adenocarcinoma at *P* < 10^−4^ (n = 296 sites), we observed highly significant enrichment for the adult blood signature at *P* = 1.2 × 10^−48^. This corresponded to an observed overlap of 66 sites having *P* < 10^−4^ in both lung adenocarcinoma and adult blood, relative to an expected overlap by chance of 5.8 sites. Lung and adult blood current smoking effect estimates for the 66 overlapping sites had Pearson correlation of 0.42 (Fig. 2A).

For overlapping sites, direction of estimated effects were concordant between lung and adult blood. Out of the 66 sites overlapping between lung adenocarcinoma and adult blood, 94% had the same direction of effect with a majority (52 of 58) of these sites hypomethylated in relation to smoking in both lung and adult blood. Among the sites with the highest absolute difference in methylation in both lung and adult blood were cg05575921 (*AHRR*) with decreased methylation in relation to smoking (lung: 9.6%, adult blood: 18.0%) and cg12803068 (*MYO1G*) with increased methylation (lung: 8.4% higher, adult blood: 6.3%). The only site where effect size was larger in blood was *AHRR* cg05575921. In all other overlapping sites, effect estimates in lung had larger magnitude (Supplementary Fig. 7). There were 20 CpGs FDR significant in adult blood, which did not meet the *P* < 10^−4^ threshold in adult blood, but did have *P* < 10^−4^ in lung. Similar to the 66 sites with *P* < 10^−4^ in both lung and blood, direction of effect was same in most sites (14 out of 20), with larger effect sizes in lung (Supplementary Fig. 8).

Among the sites overlapping between lung adenocarcinoma and adult blood, the distribution of probes across genomic regions in relation to CpG islands differed from the full array distribution (G-test of goodness of fit *P* = 4.8 × 10^−20^). We observed that 72.7% of overlapping sites were located in shore regions compared to 23.3% in the full array (*P* < 2.2 × 10^−16^), 3.1% in open sea compared to 35.7% (*P* = 6.6 × 10^−8^), and 18.2% in islands compared to 31.6% (*P* = 0.028), while the proportion of overlapping sites in shelves (6.1%) was similar to the 9.4% in the full array (*P* = 0.45).

CpG sites differentially methylated in relation to former smoking in lung adenocarcinoma were enriched for significant adult blood signals at a lower level than in lung of current smokers. CpG sites meeting the *P* threshold of 10^−4^ in lung adenocarcinoma from recent former smokers (n = 193 sites) were enriched for the adult blood smoking signature (*P* = 2.3 × 10^−23^), with 35 sites also having *P* < 10^−4^ in adult blood. Effect estimates between lung and adult blood for those 35 overlapping sites had Pearson correlation of 0.46 (Supplementary Fig. 9). Top CpGs in lung adenocarcinoma from long-term former smokers (n = 119 sites) were also enriched for significant signals in adult blood, with 12 overlapping sites (*P* = 4.1 × 10^−6^). These former smoker lung adenocarcinoma and adult blood effect estimates for those 12 sites had correlation of 0.75.

We checked whether the sites associated with smoking in both adult blood and lung adenocarcinoma were consistent across exposure groups (current, recent former, longer former) (Supplementary Fig. 10). Of the 66 sites overlapping between current smoker lung and adult blood, eight also had *P* < 10^−4^ in the lung of both categories of former smokers.

### Lung adenocarcinoma smoking DNA methylation signature is enriched for newborn blood smoking DNA methylation signature

We similarly compared the individual CpG site associations between current smoking in adenocarcinoma and maternal smoking in newborn blood. There were 3,042 CpG sites associated with maternal smoking in newborns with *P* < 10^−4^ that were represented in the lung adenocarcinoma dataset. The differentially methylated CpGs in lung were enriched for signals in newborn blood, across multiple thresholds for lung significance (Fig. 1). We again discuss results specific to the lung threshold of *P* < 10^−4^. Among these CpG sites distinguishing current smokers versus never smokers in lung adenocarcinoma at *P* < 10^−4^ (n = 296 sites), we also observed enrichment for the newborn blood smoking exposure associations (*P* = 1.2 × 10^−18^). This corresponded to an observed overlap of 24 sites, relative to an expected overlap of 2.0 sites by chance. Lung and newborn blood effect estimates for the 24 overlapping sites had Pearson correlation of 0.49 (Fig. 2B).

The overlapping sites between lung and newborn blood had the same direction of effect in 20 sites, and 16 of those sites had reduced methylation with smoke exposure in both lung and newborn blood. As in the case of adult blood and lung adenocarcinoma, cg05575921 (*AHRR*) showed reduced methylation with smoking in newborns (6.4%). Two CpGs in *MYO1G* had some of the highest differences in methylation in both newborn blood and lung: cg04180046 (11.2% in lung, 5.0% in newborn blood) and cg12803068 (8.4% in lung, 7.0% in newborn blood). In *CYP1A1*, cg18092474 had one of the largest reduction methylation in relation to smoking in lung adenocarcinoma (10.1%), and was one of the sites with largest increased methylation in newborn blood (5.2%). All 24 overlapping sites had greater magnitude of effect in lung than in newborn blood (Supplementary Fig. 7).

Sites overlapping between lung adenocarcinoma and newborn blood were distributed among genomic regions in relation to CpG islands that differed from the full array distribution (G-test of goodness of fit *P* = 7.0 × 10^−7^). We observed 70.8% of overlapping lung and newborn blood sites were in shore regions compared to 23.3% in the full array (*P* = 1.5 × 10^−7^), 4.2% in open sea compared to 35.7% (*P* = 2.7 × 10^−3^), 20.8% in islands compared to 31.6% (*P* = 0.37), and 4.2% in shelf regions compared to 9.4% (*P = *0.58). Out of the 24 sites associated with sustained smoking in newborn blood and current smoking in lung adenocarcinoma at *P* < 10^−4^, 20 (83.3%) also had *P* < 10^−4^ in adult blood.

We tested for overlap between the lung adenocarcinoma former smoker associations with the newborn blood sites. Top CpGs (*P* < 10^−4^, n = 193 sites) in lung adenocarcinoma of recent former smokers were enriched for smoking signals in newborn blood, with 24 sites overlapping with the significant newborn blood CpGs (*P* = 4.7 × 10^−23^). Effect estimates of the 24 sites had a correlation of 0.22. The newborn blood smoking signature had eight CpGs in common with top CpGs (*P* < 10^−4^, n = 119 sites) in lung adenocarcinoma from long-term former smokers (*P* = 1.6 × 10^−6^). Effect estimates of these eight sites had a correlation of 0.34. Of the 24 sites overlapping between current smoker lung and newborn blood, six also had *P* < 10^−4^ in each of the two categories of former smokers in lung. These six sites were also FDR significant in adult blood. Among those common sites, four of the CpG sites (cg19089201, cg22132788, cg12803068 and cg04180046) are annotated to *MYO1G*, cg18092474 is annotated to *CYP1A1* and cg13985198 is annotated to *SNORD46*, *RPS8*, and *SNORD38A*.

### Sensitivity analyses

As a sensitivity analysis, enrichment tests were also performed for blood thresholds of FDR < 0.05 as opposed to *P* < 10^−4^. In adult blood 18,126 CpGs met this threshold, and in newborn there were 5,936 CpGs. Patterns of enrichment were similar to those seen using *P* < 10^−4^ significance criteria in blood (Supplementary Fig. 11). Findings were also robust to varied *P* thresholds in lung adenocarcinoma CpGs. We allowed *P* cutoffs in lung associated CpGs to vary from 1.0 to 10^−10^ (Fig. 1). When also using FDR < 0.05 cutoffs in lung results, 66 CpGs met the criteria in lung of current smokers, with 30 sites overlapping with FDR significant adult blood CpGs (*P* = 1.2 × 10^−24^) and 14 sites overlapping with newborn blood CpGs (*P* = 1.6 × 10^−13^). In lung of recent former smokers (≤15 years) 19 CpGs were FDR significant, with 14 overlapping with the adult blood signal (*P* = 2.3 × 10^−16^) and 10 overlapping with newborn blood signal (*P* = 1.1 × 10^−14^). In lung of long-term former smokers (>15 years) 14 CpGs were FDR significant, with 4 overlapping with adult blood signal (*P* = 1.8 × 10^−3^) and 3 overlapping with newborn blood signal (*P* = 7.2 × 10^−4^). We analyzed enrichment of adult blood signatures in 10,000 random permutations using *P* < 10^−4^ cutoffs. In none of the permutations was overlap of top CpGs with adult blood signals equal or more extreme than what we observed. The lowest *P*-values in any permutation were 9.0 × 10^−5^, 4.6 × 10^−4^, and 2.6 × 10^−5^ in current smokers, recent former smokers, and long-term former smokers, respectively.

**Figure 3:**
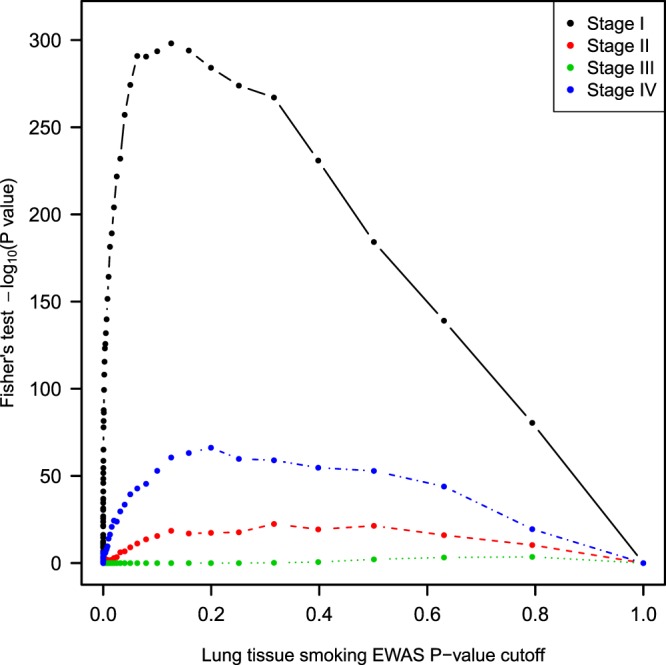
Adult blood smoking signal enrichment in lung stratified by cancer stage. Fisher’s enrichment test results comparing overlap of DNA methylation sites associated with current smoking in lung adenocarcinoma and sites in adult blood (*P* < 10^−4^), stratifying samples by cancer stage. The smoking signature in blood is most strongly enriched in stage I samples.

When stratifying by cancer stage the smoking signal in blood is enriched most strongly in the stage I samples (Fig. 3). Using the same *P* < 10^−4^ cutoffs in the primary analysis, top CpGs for current smoker versus never smoker for stage I cancers had 71 sites overlapping with the adult blood signal compared to 10 expected CpGs (*P* = 1.1 × 10^−37^). In other stages, enrichment is small (stage II *P* = 0.14, stage III *P* = 1.0, stage IV *P* = 0.04), with expected overlaps of less than two CpG sites. Very few sites in later stages met the *P* < 10^−4^ cutoff. In the stage I group, there were 513 such CpG sites, but in stages II through IV there were 34, 6, and 64, respectively.

### Bladder carcinoma smoking associated sites

We ran the same tests for enrichment of blood signals in TCGA bladder carcinoma data (N = 372). There were only two stage I cancers after sample filtering (Supplementary Table 2), which we combined with the stage II group when running regression models. In bladder carcinoma tissue, DNA methylation was less strongly associated with smoking than in lung adenocarcinoma. No CpG sites were FDR significant (full results in Supplementary Dataset 2). Enrichment of smoking-associated blood signals was low to nonexistent (Supplementary Fig. 12). In bladder carcinoma, 43 CpG sites had *P* < 10^−4^ when comparing current smokers to never smokers. One of those sites overlapped with the adult blood signal (Fisher’s test *P* = 0.57), and none with the newborn signal (Fisher’s test *P* > 0.99). For recent former smokers, 52 CpG sites had *P* < 10^−4^, with one overlapping with the adult blood signal (Fisher’s test *P* = 0.64), and two with the newborn blood signal (Fisher’s test *P* = 0.05). In long-term former smokers, 39 CpG sites had *P* < 10^−4^ with four overlapping with the adult blood signal (Fisher’s test *P* = 0.007), and none with newborn blood signal (Fisher’s test *P* > 0.99). None of the top (*P* < 10^−4^) CpGs in lung overlapped with top CpGs in bladder. Of the top 43 CpGs in bladder of current smokers, 20 had the same direction of effect in lung. Examining the 66 CpGs that overlapped between lung of current smokers and adult blood, direction of effects in adult blood, lung and bladder of current smokers was the same in 54 out of 66 sites.

### Gene ontology of sites uniquely associated with smoking in lung and not blood tissues

We investigated whether lung-specific DNA methylation changes in response to smoke exposure could provide insight to the biology of lung adenocarcinoma. Although the differentially methylated CpGs in lung adenocarcinoma were enriched for smoking signals in blood, we also identified several CpG sites in lung adenocarcinoma that were not implicated in either blood meta-analyses, both of which had a much larger sample size than the present study. There were 36 sites in current smoker lung that were FDR significant, 4 sites in former smokers (≤15 years), and 10 sites in former smokers (>15 years), that were not also FDR significant in adult blood or newborn blood (Supplementary Dataset 3).

To perform a pathway analysis on lung-specific sites, we used the relaxed threshold of *P* < 10^−4^ to include a sufficient number of sites. The CpG sites that reached *P* < 10^−4^ in lung adenocarcinoma of current smokers were annotated to 224 genes, and of those 105 were not implicated either the meta-analyses in blood by a CpG with *P* < 10^−4^. No biological pathways these 105 genes are involved in were enriched at an FDR significant level. The top pathways (*P* < 0.01) include mRNA catabolic processes, protein targeting, angiogenesis, and translation (Supplementary Table 3). Top pathway findings were largely driven by ribosomal subunit genes (*RPL14*, *RPS16*, *RPS23*, *RPS27A*) and microRNA genes (*MIR17*, *MIR18A*, *MIR19A*, *MIR19B*, *MIR20A*) involved in multiple pathways, and both sets of genes were involved in the translation pathway.

As a comparator, we also performed pathway analysis on overlapping lung-blood sites. A total of 52 genes were implicated by CpG sites that had *P* < 10^−4^ in lung of current smokers and in both adult and newborn blood. Several FDR significant gene ontologies for the common lung adenocarcinoma and blood smoking genes included many of the same or similar pathways, such as protein targeting to membrane, mRNA catabolic process, and translation (Supplementary Table 4). Ribosomal subunit genes were also involved in all of the top pathways. Genes found in both the top lung sites and blood sites include *AHRR, CYP1A1, CYP1B1*, *MYO1G*, several small nucleolar RNA genes, ribosomal subunit genes, and others (Supplementary Table 5). In 100 permutations selecting different random subsets of 60 smoking associated sites in lung, more than 80% of top pathways (*P* < 10^−3^) of the permutations contained protein targeting to membrane and translation related pathways.

## Discussion

Tobacco smoking or exposure to tobacco smoke has been consistently associated with altered DNA methylation in blood measured across the life course^3,5^. Many of the genome-wide significant sites in lung adenocarcinoma mirror those found in blood. In lung adenocarcinoma of current smokers, genome-wide significant CpGs annotated to multiple small nucleolar RNA genes, ribosomal subunit genes (*RSP*s), myosin immunoglobulin (*MYO1G*), and zinc finger protein 28 (*ZFP28*). Both CpGs annotated to *RPS8*, cg13985198 and cg18806997, were hypomethylated in lung adenocarcinoma and also significantly hypomethylated in adult blood^5^ and cg13985198 was additionally significantly hypomethylated in newborn blood^3^. Both genome-wide significant CpGs in *RPS18* (also mapping to vacuolar protein sorting 52 (*VPS52*)), cg07362537 and cg12086028, were also significantly hypomethylated in both adult and newborn blood^3,5^. Significant CpGs in *MYO1G*, cg22132788 and cg19089201, were hypermethylated and also found to be hypermethylated in adult and newborn blood^3,5^. In recent former smokers, there were multiple genome-wide significant CpGs in *MYO1G* and cytochrome p450 family genes *CYP1A1* and *CYP1B1*. Interestingly, all three of these genes were differentially methylated at genome wide significance in blood in relation to smoking in both newborns and adults^3,5^. The genome-wide significant CpG annotated to *HIPK2* (cg03224163) was also found in adult blood^5^. There were two CpGs associated with longer former smoking status at a genome-wide level annotated to cadherin EGF LAG Seven-Pass G-Type Receptor 3 (*CELSR3*), a gene identified previously in adult blood of former smokers relative to never smokers^5^. The other significant CpG site, cg22132788, was mapped to *MYO1G* and was one of the same sites found in both recent former smokers and current smokers.

Comparing systematically at multiple cut points, we identified DNA methylation alterations in lung adenocarcinoma associated with smoking and found both concordance, and discordance, between these smoking associated DNA methylation alterations and those previously reported in newborn and adult blood samples. Interestingly, when stratifying analyses by recency of smoking cessation, the highest enrichment for smoking-related DNA methylation changes previously observed in adult and newborn blood were seen in differentially methylated CpGs in lung adenocarcinoma of current smokers. These results support existing evidence that most DNA methylation alterations related to tobacco smoke exposure are attenuated with quitting time, and that the effects of tobacco smoke exposure in lung cancer tissue are not permanently mitotically heritable. These findings provide evidence that suggest there may be a consistent smoking-associated DNA methylation signature across tissues. We found that these smoking-associated signatures across tissues had larger effect sizes in lung, except for cg05575921 in *AHRR*; this CpG shows the strongest association in nearly all studies of smoking in relation to blood methylation. Out of 296 CpGs in current smokers meeting the *P* < 10^−4^ cutoff 66 overlapped with the adult blood signature, leaving a large percentage (78%) of those sites as unique smoking DNA methylation associations in lung adenocarcinoma tissue, suggesting there may also be a significant tissue specific component in epigenetic alterations in response to tobacco smoke. Relatively low overlap in methylation patterns may be a reality of differing tissue types. In blood and brain, only 7.9% of CpG sites on the 27k array^21^ and 9.7% of CpGs on the 450 k array^22^ were informative of both tissues.

We did not find the same smoking signatures in the top TCGA bladder carcinoma results. Overall, methylation had weaker association with smoking in bladder cancer than in lung cancer. This is perhaps expected because lung cells are exposed to tobacco combustion products both directly via inhalation and via the blood stream. In contrast, bladder cells experience only blood borne exposure. The greater level of exposure to smoking in lung is reflected in the effect sizes as well, where lung generally had larger smoking-related methylation differences than blood and bladder. Further, the two genes first identified as differentially methylated in relation to smoking, *AHRR* and *CYP1A1*, play opposing roles in the aryl hydrocarbon receptor pathway which has a major role in the response to polyaromatic hydrocarbons such as are created when cigarettes are burned. Unlike with cigarettes, use of snuff is not associated with differential methylation in blood^23^, which is additional evidence of the importance of compounds created from burning. Lung cells are directly exposed to the burning, but the bladder is not. Second, smoking also has a larger impact on risk of cancer in lung. Among smokers, there is a 10 fold increase in risk of lung adenocarcinoma (45 fold increased risk in squamous and small cell lung cancer)^24^, while in bladder cancer smokers have a three fold increase in risk^25^. Lastly, our lung adenocarcinoma dataset was primarily derived from tumors with cancer stage I, while the bladder carcinoma dataset had more tumors at later stages. Genomic instability is expected throughout cancer progression, which may diminish previous environmental signals. Thus, our results that blood methylation more closely reflects smoking associated changes in lung than in bladder is not surprising. However, it is worth noting the sites that did overlap between adult blood and lung largely had the same direction of effect in bladder. Smoking may have a more pronounced effect on DNA methylation in lung, and consequently we had more power to detect the large effect sizes in lung compared to smaller effects in bladder.

These results, however, should be interpreted in light of the high rates of epigenetic dysregulation in tumors^26^, where cells are rapidly cycling and there is persistent dysregulation of the epigenetic machinery. Epigenetic alterations in lung cancers could be caused directly by tobacco smoke exposure or reflect epigenetic changes as a result of cancer progression. There is a possibility that non-diseased, rather than cancerous, lung tissue from smokers would more closely reflect the epigenetic signature associated with tobacco smoke exposure in blood. For example, a recent study of epigenetic alterations identified seven significantly differentially methylated CpG sites in non-tumor lung tissue from smokers, five of which were also previously found to be differentially methylated in smoker’s blood^7^. Possible signs of DNA methylation alterations with cancer progression are observed in our enrichment results when stratified by cancer stage. Blood smoking signals were most strongly enriched in stage I samples. These findings, suggest that our enrichment is largely driven by smoking effects in stage I samples. Part of this pattern is likely due to progressively smaller samples sizes in later stage samples. While the greater sample size for lower stage cancers might explain some of the difference, it may also be possible that blood smoking signatures that would have been observed were masked by cancer progression related changes. This may also explain lack of blood signal enrichment in bladder cancer, where there were very few stage I samples, and more late stage cancers.

We, and others, have identified smoking associated DNA methylation alterations in genes which have been identified to have functional roles relative to lung cancer initiation and progression. Epigenetic alterations may drive the formation of lung cancer by sensitizing cells to *KRAS* mutation^27^. *AHRR* was hypomethylated with smoking in both lung cancer and blood, and hypomethylation of *AHRR* is associated with future lung cancer after adjustment for smoking^28^, as well as low lung function, decline in lung function, and respiratory symptoms^29^. While this may be explained, in part, by measurement error in self-reported smoking combined with *AHRR* methylation being an excellent quantitative biomarker of lifetime smoking behavior that captures this exposure better than questionnaires^30^, smoking related reduced methylation in *AHRR* could play a role in pathogenesis. While a role for *MYO1G* in lung carcinogenesis has not been established, we also identified concordant methylation of CpG sites in *MYO1G* associated with smoking in lung cancer and both newborn and adult blood. Interestingly, siRNA knockdown of *MYO1G* in multiple cancer cell lines increased cell death and decreased autophagic flux, a process dysregulated in many human disorders^31^. Whether *MYO1G* methylation in the lung is simply a biomarker of smoke exposure or has a functional role in cancer development remains to be determined. Intriguingly, while *CYP1A1* is hypermethylated relative to smoke exposure in newborn blood, we found a CpG site upstream of the *CYP1A1* transcription start site to be hypomethylated in lung cancer adenocarcinoma relative to smoking. *CYP1A1* polymorphisms have been linked to lung cancer risk, particularly when in combination with tobacco smoke^32–34^, pointing to this gene’s important role in tobacco smoke toxicant metabolism and lung cancer etiology. Interestingly, the promoter of *CYP1A1* in normal lung tissue has been found to be hypermethylated in smokers^35^, similar to the findings reported newborn blood, but not in lung cancer tissue. However, the *CYP1A1* annotated CpGs hypomethylated in lung adenocarcinoma were also observed to have decreased methylation in adult blood findings.

Besides *AHRR, MYO1G*, and *CYP1A1*, highlighted for having large effect sizes, several other genes implicated by CpGs overlapping between lung and blood are also cancer related. *GAS5* had several mapped CpGs associated with smoking in adult blood, newborn blood, and in lung adenocarcinoma (including the genome-wide significant cg16290996). Downregulation of *GAS5* has been observed in lung adenocarcinoma and lower expression is associated with increased tumor size, poor differentiation, and advanced pathological stage^36^. Similarly, reduced GAS Antisense RNA 1 (*GAS5-AS1*) expression is associated with larger tumor size and tumor metastasis^37^. Many small nucleolar RNA genes had related CpGs in lung and blood differentially methylated in association with smoking. Small nucleolar RNAs are involved in regulation of several cell processes and may be involved in tumorigenesis^38^. *SNORD78*, which mapped to multiple smoking-associated sites in lung (including genome-wide significant cg16290996) and blood, is upregulated in non-small cell lung cancer tissue and suppresses proliferation when inhibited^39^. *SNHG1* also has multiple CpGs associated with smoking and lung and blood, including three which achieved genome-wide significance in lung (cg02905828, cg09345320, cg27033919). Inhibition of *SNHG1* suppressed non-small cell lung cancer proliferation *in vitro* and *in vivo*^40^. *HIPK2*, which we previously mentioned as having significant smoking associated CpGs in lung and adult blood, is involved in regulation of apoptosis and angiogenesis, and its activity inhibits tumor growth^41^. Several of the genes with mapped CpGs that overlapped between lung and blood have been previously shown to have smoking associated altered gene expression in whole blood, namely *CYP1B1*, *C6orf48*, *NME1-NME2*, *ZNF773*, and several ribosomal subunit proteins (such as *RPL8* and *RPS6*)^42^. Additionally, a *C6orf48* related SNP was one of many SNPs associated with lung carcinoma risk in a genome-wide association study^43^.

Smoking-related DNA methylation at CpGs not implicated in blood were also annotated to genes potentially related to lung cancer. Some of the lung unique CpGs were annotated to genes in which other CpGs did overlap with blood signals, such as *SNHG1* and *C6orf48*. In pathway findings, top results were in part largely driven by a set of microRNA genes. *MIR17*, *MIR18A*, *MIR19B1*, and *MIR20A* are part of the miR-17–92 cluster on chromosome 13 that is upregulated in lung cancer cell lines, and are involved in repression of proliferation inhibition and apoptotic agents^44^. Ca2 + /calmodulin-dependent protein kinase kinase 2 (*CAMKK2*) was overexpressed in hepatic^45^ and gastric^46^ cancer cells, and its inhibition slowed tumor growth. Determining whether the DNA methylation markers associated with smoking in lung cancer are drivers or passengers of the carcinogenic progression will be essential to understand the clinical impact of these alterations.

The present study had several weaknesses. Despite a large number of lung adenocarcinoma cancer cases analyzed here (n = 390), the power is much lower than in the two larger meta-analysis of adult and newborn smoking. The smoking-associated DNA methylation changes in the lung were not as numerous as those in adult and newborn blood which is likely to be consequence of the weaker power. Differences in study populations may also impact comparability of the CHARGE adult blood and the TCGA lung adenocarcinoma. CHARGE participants, recruited from various population-based cohorts, were on average slightly younger than those in TCGA, consistent with the increase in cancer risk with age. The average ages of current smokers, former smokers, and never smokers in CHARGE were 57.7, 64.8, and 61.2 years^5^. In TCGA, there was an average age of 61.4 for current smokers, 63.9 for recent former smokers, 70.5 for long term former smokers, and 65.6 for never smokers. Second, TCGA lung tissue observations were derived from tumor tissues, which introduces the possibility that apparent smoking associated DNA methylation changes were a consequence of disease. Tumorigenesis is frequently associated with *de-novo* DNA methylation changes with enormous selection pressures on cells^47^. Our observations demonstrate either smoking associated changes survive tumorigenesis in lung adenocarcinoma, or they are recapitulated in the disease process, both suggesting they are of high biological importance in this tissue. Epigenetic changes in tumor cells during cancer progression may also erase smoking-associated signatures mirroring those seen in blood that would otherwise been observed. This issue could be explored by repeating the current study in non-diseased lung tissue as paired smoking status and DNA methylation measures become available. Further, tumors are heterogeneous mixtures of cell types, potentially also containing blood cells, each of which will have its own epigenetic profile. Recent work has shown that even within blood, some of the methylation changes associated with smoking are cell type specific^48^. Studies would be strengthened by the concurrent measurement of DNA methylation profiles of blood and a target tissue in the same individual. Determining whether smoking related epigenetic alterations in lung cancer are persistent across all cells or are relegated to individual cell types, with functional regulatory roles, will be an exciting future direction of research.

Integrated analyses of exposure related epigenomic signatures across tissue types represents a powerful approach to disentangle systemic and tissue specific alterations due to exogenous exposures. Here, for example, we identified a highly significant overlap of altered CpGs in lung tumor and blood due to smoking, providing support for the hypothesis that tissue types typically assayed by epidemiologists, such as blood, can provide relevant information about epigenetic alterations in target tissues, such as lung. The NIEHS Toxicant Exposures and Responses by Genomic and Epigenomic Regulators of Transcription (TaRGET) II consortium is currently formally testing this hypothesis across a range of exposures, tissue types, and epigenomic marks in mice^49^. We also, however, identified many CpG sites altered related to smoking in lung cancer tissue, which were not previously reported in studies in blood (Supplementary Dataset 2). These CpGs may represent unique effects in lung. If these CpGs were also differentially methylated in blood, the higher powered meta-analyses would likely have discovered them as well. However, given the small sample size for a genome-wide study and lack of validation, there is considerable risk of false positives. When evaluating overlap using a more stringent FDR < 0.05 cutoff in lung, a higher portion of CpG sites meeting the threshold overlapped with blood signals. Furthermore, pathway analyses identified that these lung cancer specific alterations were enriched in genes and pathways involved in RNA catabolism, protein targeting, and transcription, which were very similar to pathways identified in sites overlapping with blood and not unique to lung. The permutation results, selecting subsets ignoring overlap or uniqueness, contained similar pathways, further suggesting pathways in overlapping and lung unique CpGs reflect pathways dominating in the overall smoking-associated DNA methylation patterns in lung. Many ribosomal subunit proteins were represented in the top lung adenocarcinoma findings, many of which were also found in blood. The prevalence of these genes in pathway findings may be related to cancer, as ribosomal proteins often show increased expression in such cases^50^. Understanding the tissue specific epigenomic signatures related to exposure may be able to identify etiologic agents in tumor development, critically impacting prevention and treatment.

In conclusion, our results suggest a subset of smoking-related methylation signals in blood reflect signals in lung. Smoking-related DNA methylation signals in lung adenocarcinoma had highly significant overlap with those previously found in blood of newborns and adults. Methylation patterns with smoking in bladder cancer, on the other hand, did not overlap with blood and lung. While we found many methylation signals are common across some tissues, many other changes in methylation associated with smoking may be tissue specific. Our findings support the value of methylation biomarkers assessed in blood for providing insight into exposure effects in lung tissue. However, a measure of caution is advised in interpreting our results, given modest sample sizes, possible effects of epigenetic dysregulation in tumors, and cell type differences, both within and across tissues. Further studies should examine cross tissue overlap in non-diseased lung, as well as in other tissues.

## Supplementary information

Supplement

Supplementary Dataset 3

Supplementary Dataset 1

Supplementary Dataset 2

## Data Availability

The Cancer Genome Atlas data that support the findings of this study are publicly available through the National Cancer Institute Genomics Data Commons Portal (https://portal.gdc.cancer.gov/). The findings in newborn blood^3^ and adult blood^5^ are available in the supplementary material for the respective manuscripts.
