# Supplementary material for: DNA methylation signature of smoking in lung cancer is enriched for exposure signatures in newborn and adult blood
Source: Sci Rep. 2019 Mar 14;9:4576. doi: 10.1038/s41598-019-40963-2 (PMC6418160; doi:10.1038/s41598-019-40963-2)
Supplement: Supplementary file 1 — Supplement [file 41598_2019_40963_MOESM1_ESM.docx]

**DNA methylation signature of smoking in lung cancer is enriched for exposure signatures in newborn and adult blood.**

Kelly M. Bakulski, John Dou, Nan Lin, Stephanie J. London, Justin A. Colacino

**Supplementary Information**

| **A.**  **** | **B.**  **** |
| --- | --- |
| **C.**   | **D.**  **** |

**Supplementary Figure 1.** Flow charts showing criteria for dropping The Cancer Genome Atlas samples and Illumina 450k DNA methylation probes from analysis. **A.** Sample quality control in lung adenocarcinoma. **B.** DNA methylation probe quality control in lung adenocarcinoma. **C.** Sample quality control in bladder carcinoma. **D.** DNA methylation probe quality control in bladder carcinoma.

| **A.** 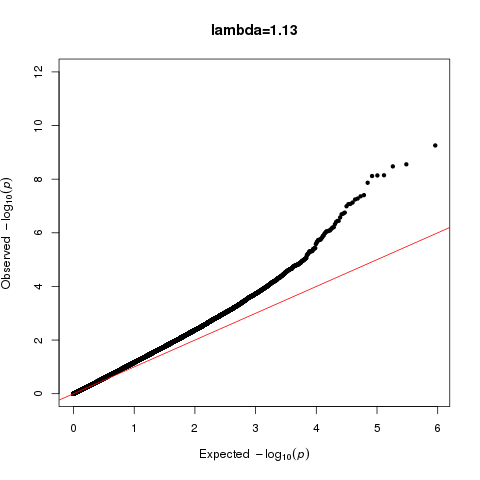 | **B.** 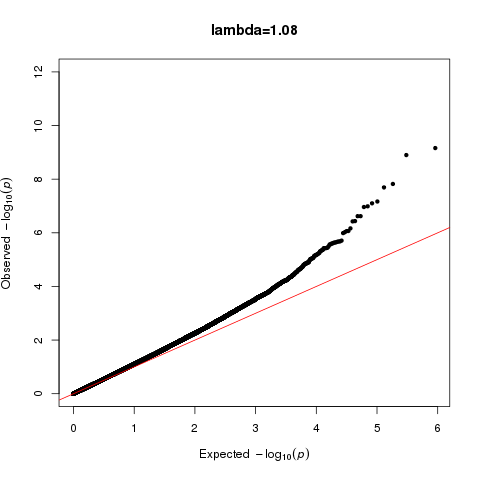 |
| --- | --- |

**Supplementary Figure 2.** Expected versus observed *P*-value distribution in lung adenocarcinoma samples from The Cancer Genome Atlas (TCGA) for the association between DNA methylation and current versus never smoking status. **A**. Primary model was adjusted for age, 10 ancestry principal components, batch, cancer stage, and sex. **B**. Sensitivity model was adjusted for age, 10 ancestry principal components, batch, cancer stage, sex, and 10 surrogate variables.

| **A.** 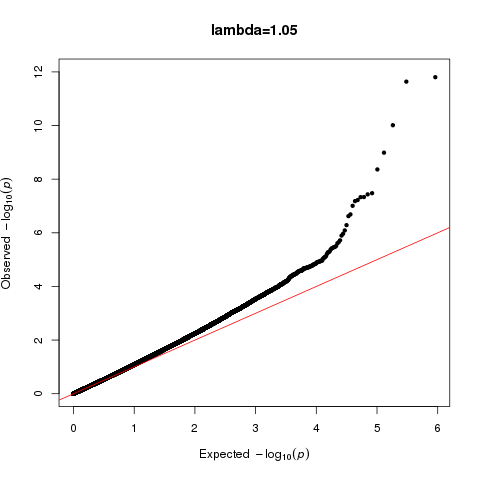 | **B.** 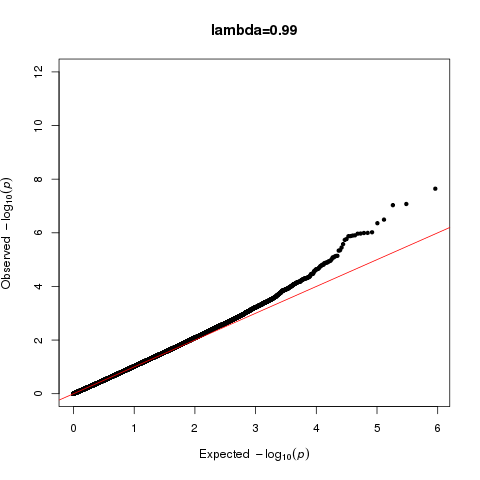 |
| --- | --- |

**Supplementary Figure 3.** Expected versus observed *P* value distribution in lung adenocarcinoma samples from The Cancer Genome Atlas (TCGA) for the association between DNA methylation and former versus never smoking status. Models were adjusted for age, 10 ancestry principal components, batch, cancer stage, and sex. **A.** Recent former smokers that quit within the last 15 years. **B.** Longer term former smokers that quit smoking more than 15 years ago.


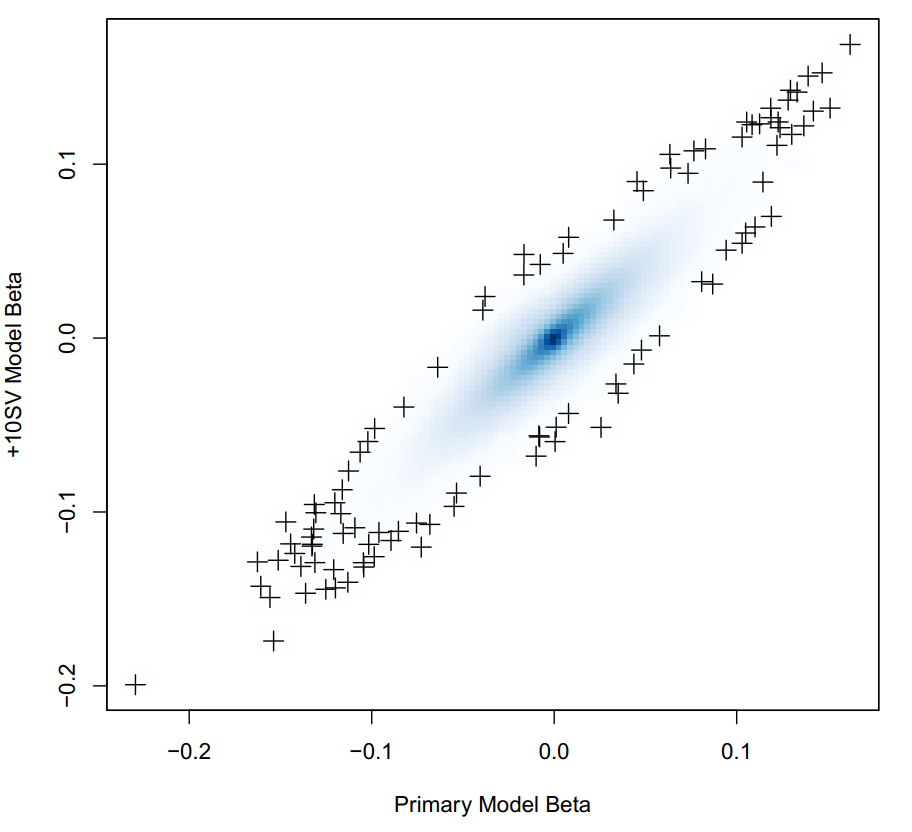


**Supplementary Figure 4**. Smooth scatter plot of effect estimates for DNA methylation in current smokers vs never smokers from the primary model (adjusted for age, 10 ancestry principal components, batch, cancer stage, and sex) and from the surrogate variable model (10 surrogate variables added). 100 points in least dense regions are plotted.


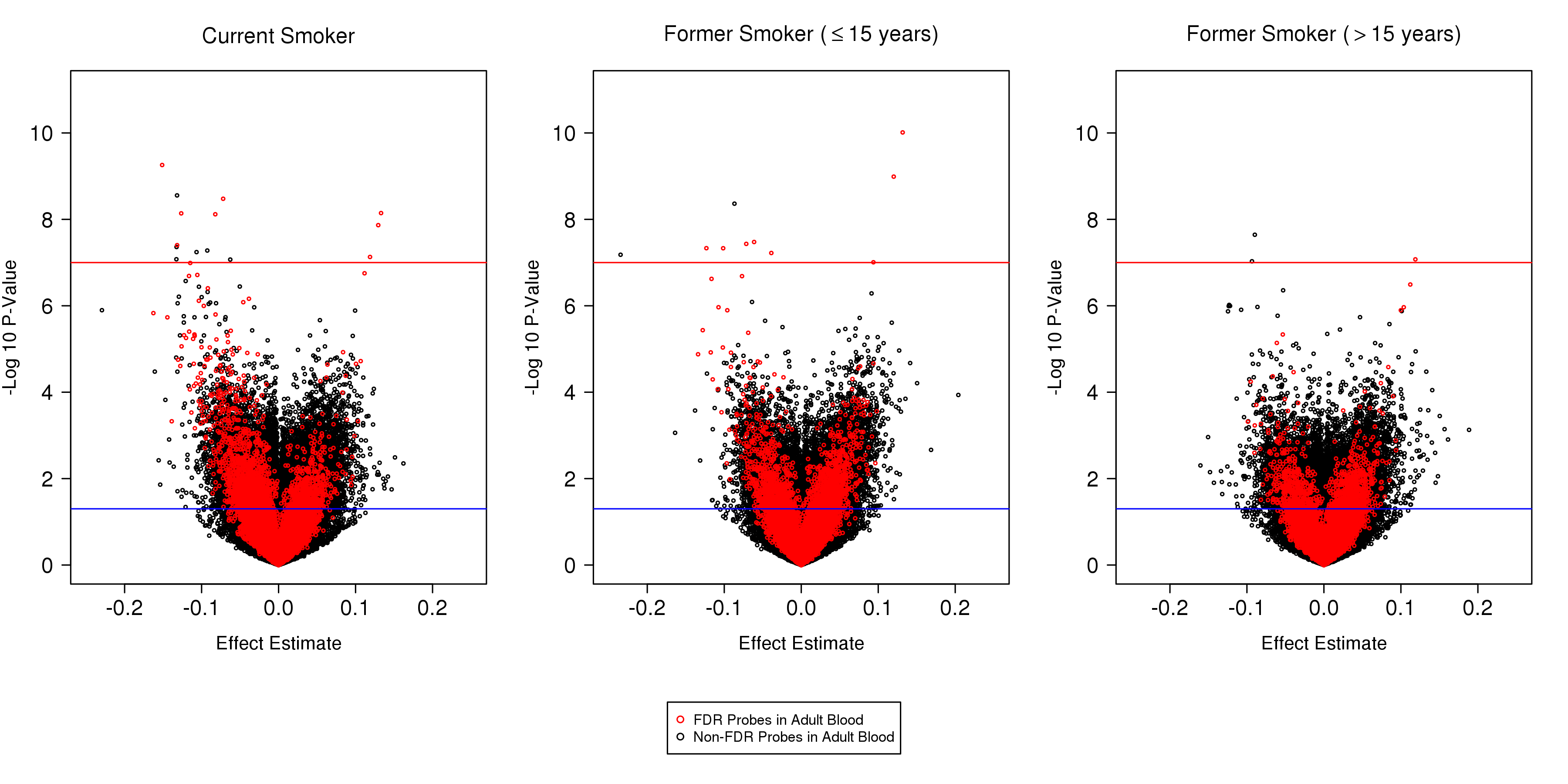


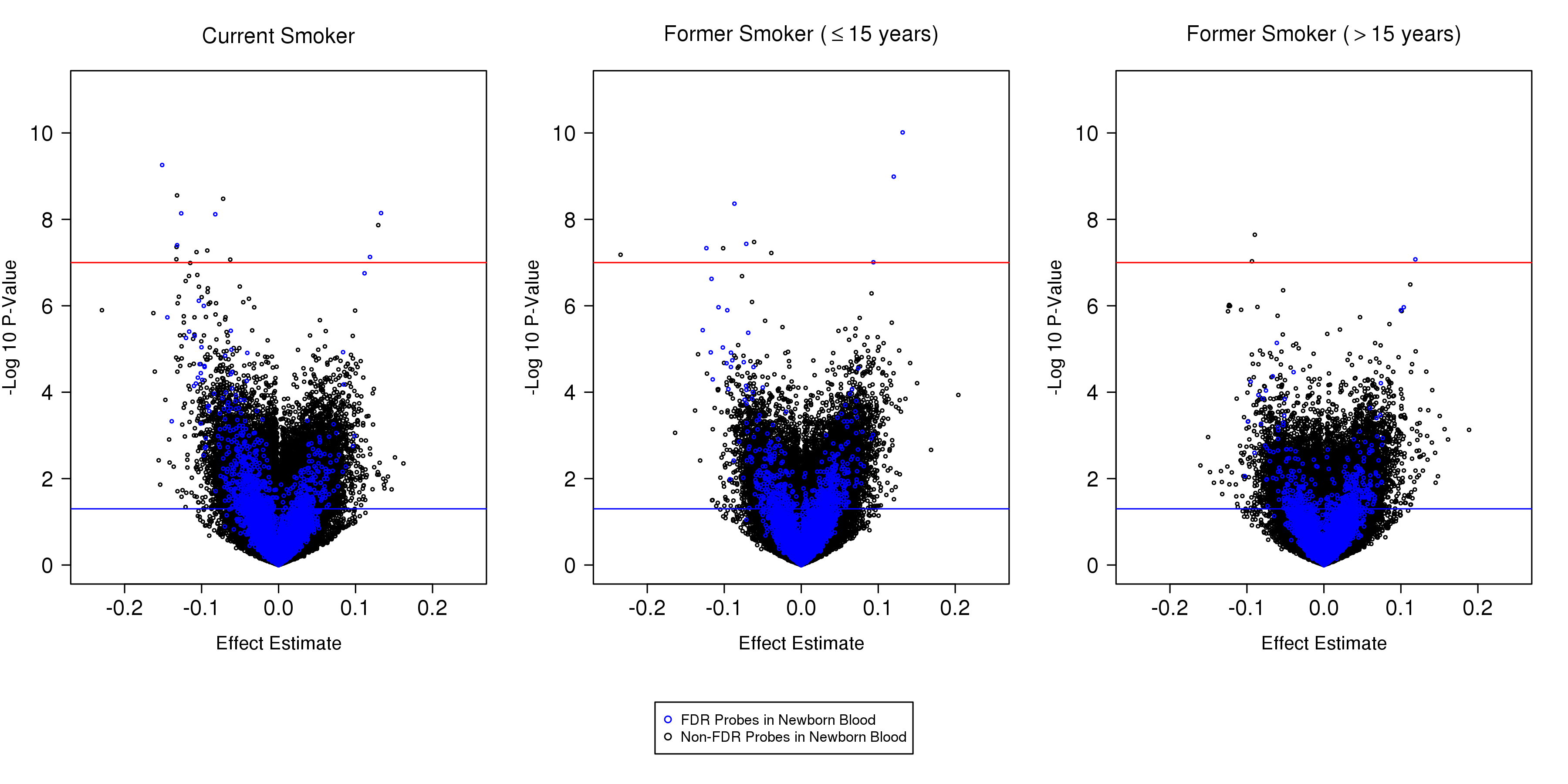


**Supplementary Figure 5.** Volcano plots of per CpG effect estimates of smoking on DNA methylation in lung adenocarcinoma samples and -log_10_(*P*-values). Blue line is at a *P*=0.05 level, and red line is drawn at the *P*=10^-7^ level. In upper row plots probes that were FDR<0.05 in the adult blood meta-analysis are plotted in red (N=17,878), and in the bottom row of plots probes FDR significant in newborn blood meta-analysis are plotted in blue (N=5,924). Black points are probes not overlapping with blood signatures.

| **A.** 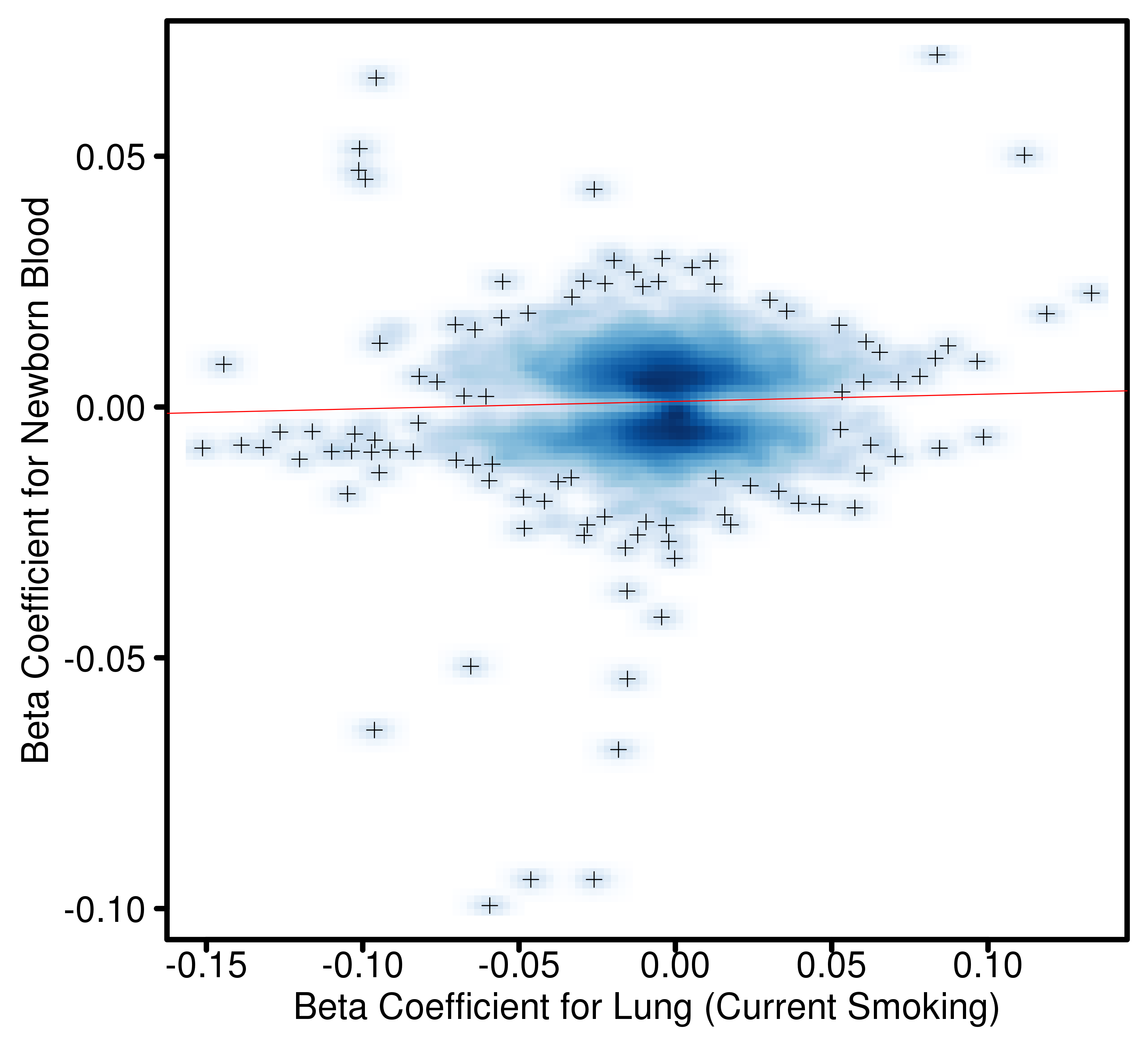 | **B.** 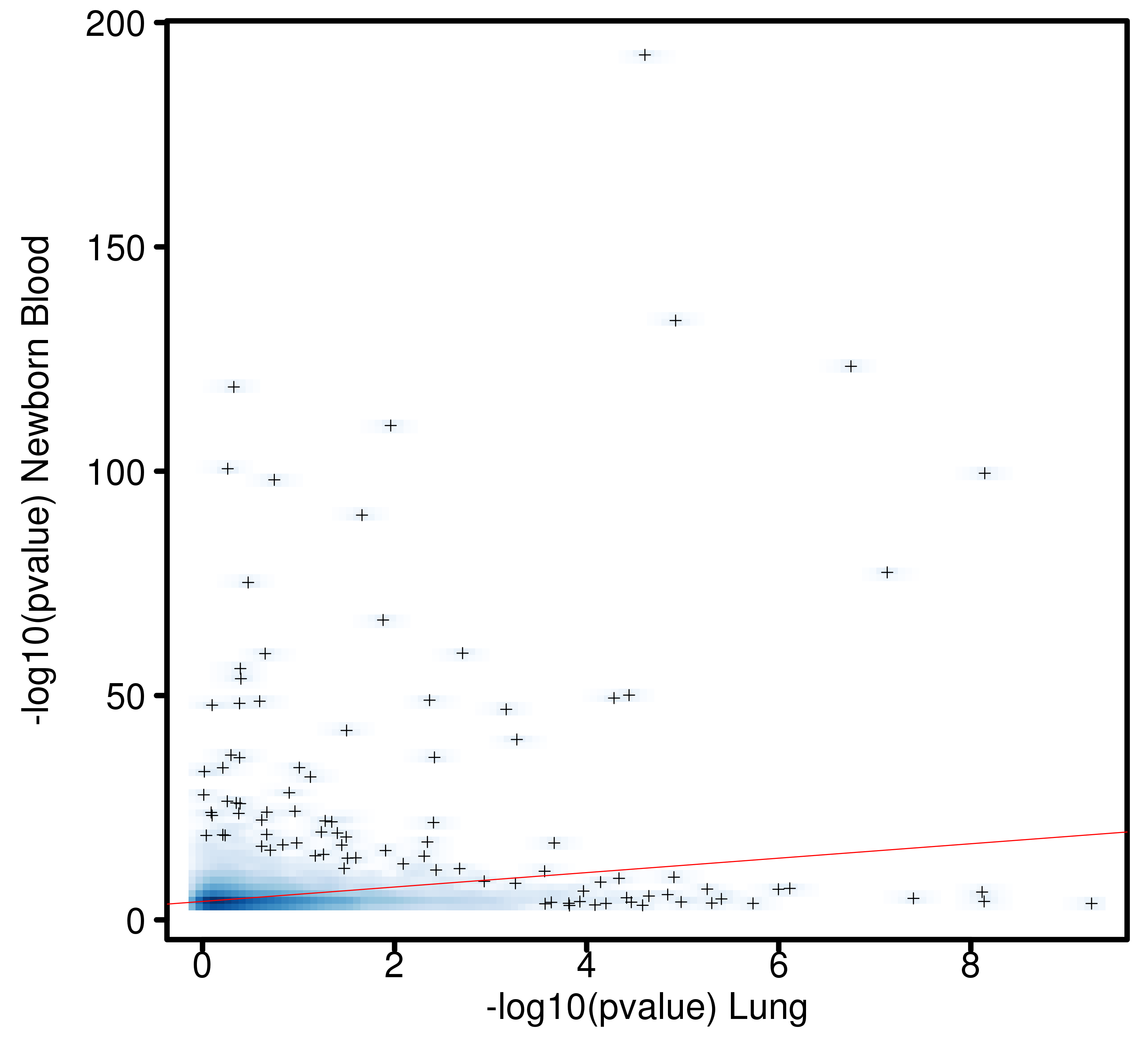 |
| --- | --- |
| **C.** 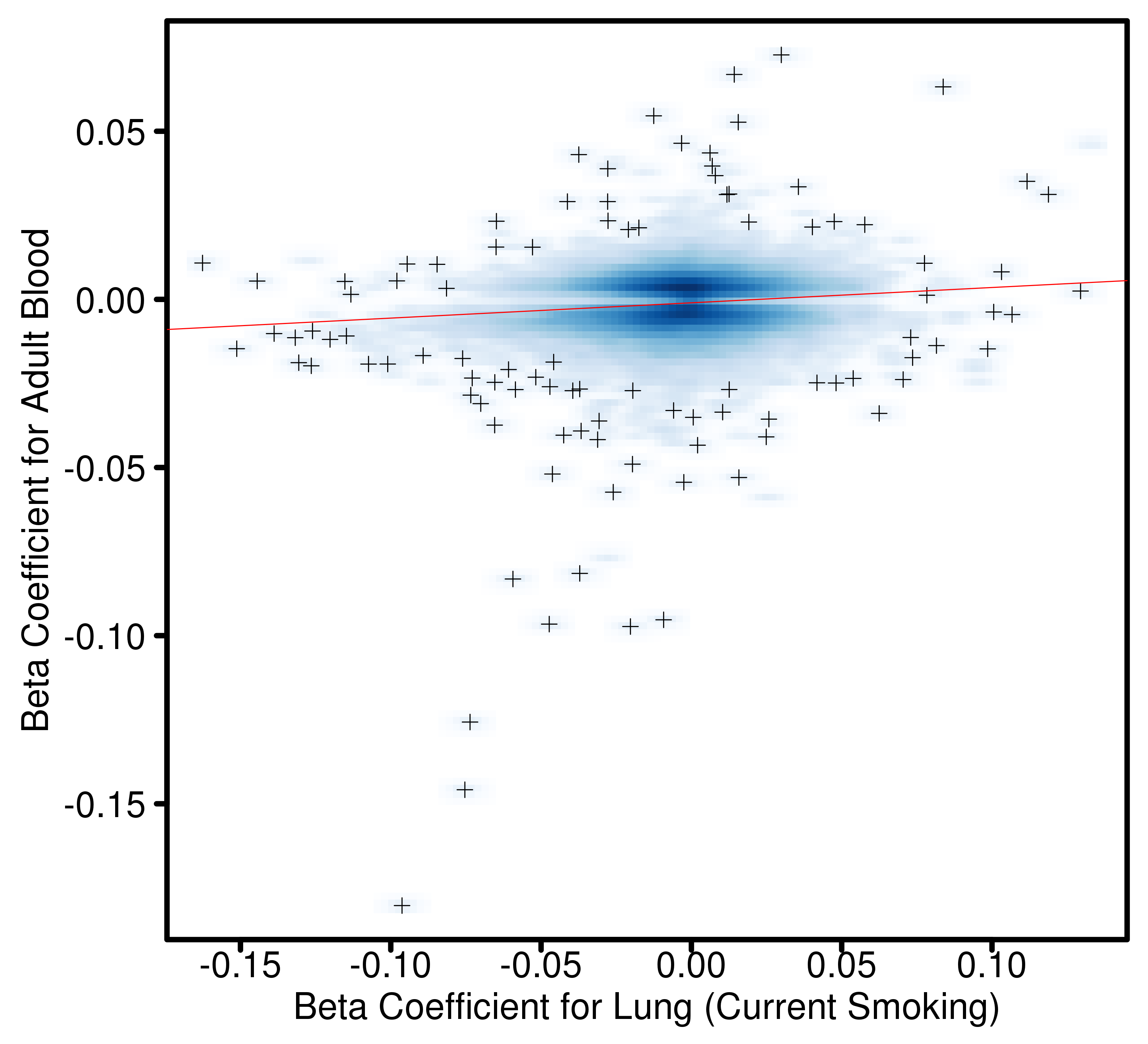 | **D.** 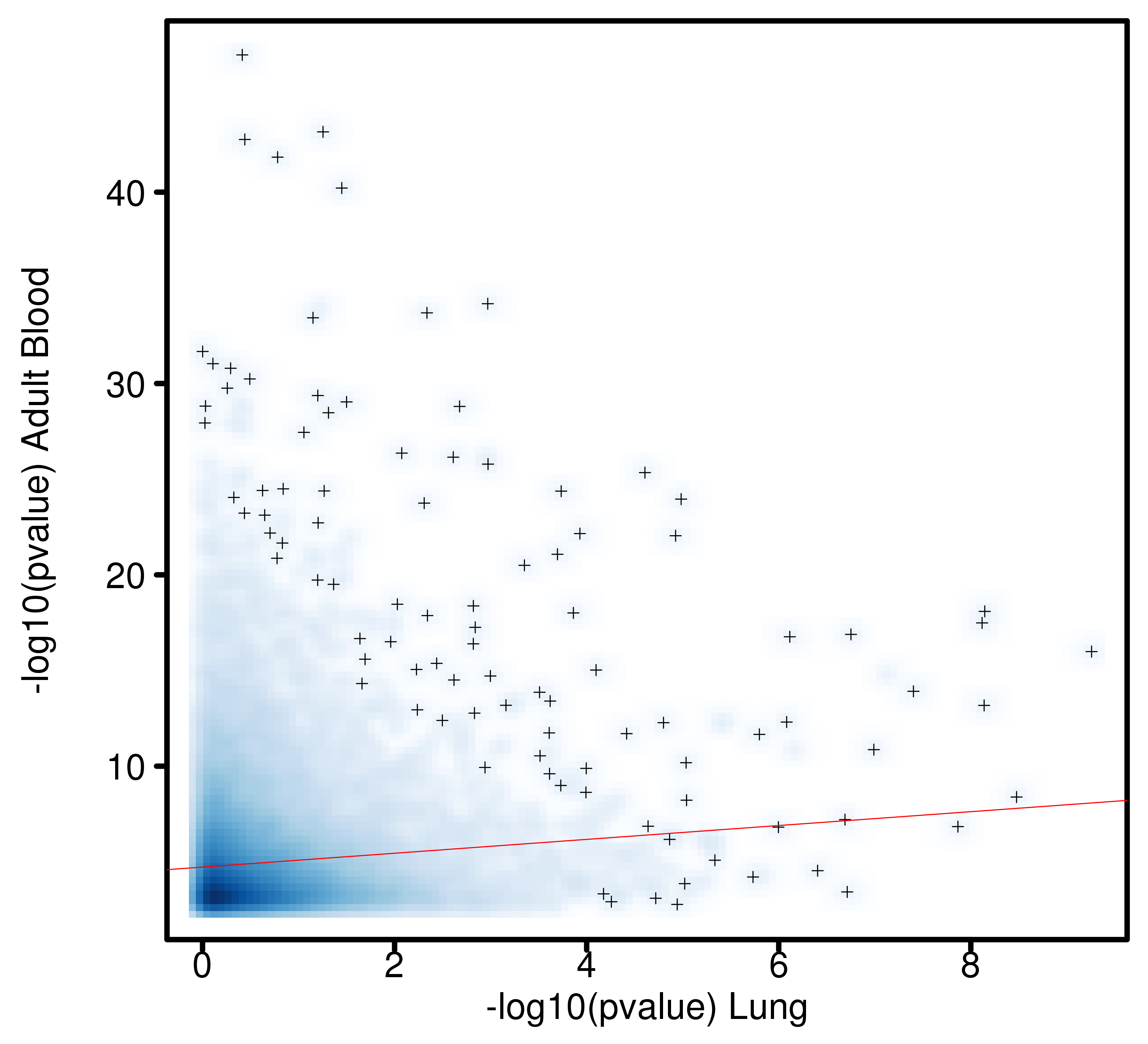 |

**Supplementary Figure 6.** Scatterplots showing the relationships between DNA methylation effect estimates (**A, C**) and –log_10_(*P*-values) (**B, D**) for smoking in newborn blood and lung adenocarcinoma tissue samples (**A, B**) and for smoking in adult blood and lung adenocarcinoma samples (**C, D**).

| **A.** | **B.** |
| --- | --- |
| 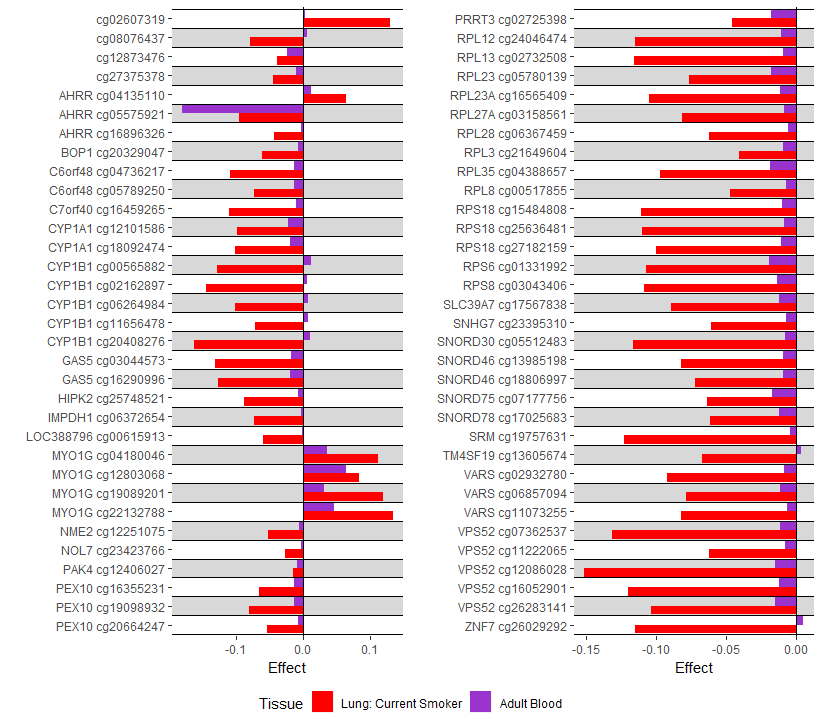 | 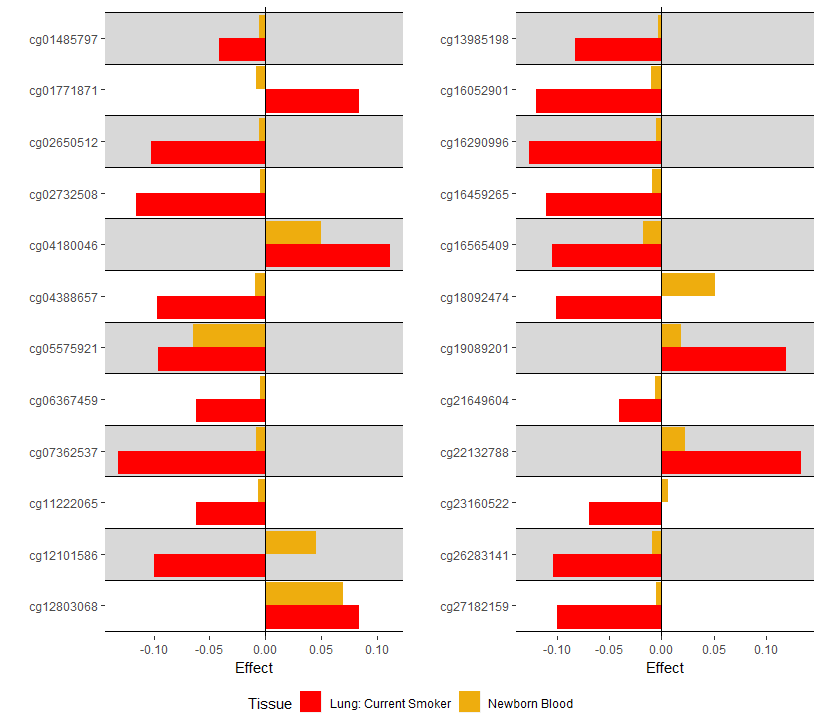 |

**Supplementary Figure 7.** DNA methylation is associated with smoking status (current versus never) in lung adenocarcinoma tissues from The Cancer Genome Atlas (TCGA) and in adult or newborn blood. **A.** Comparison of current smoking DNA methylation effect sizes for CpGs with *P*<10^-4^ in lung adenocarcinoma and in adult blood from Cohorts for Heart and Aging Research in Genomic Epidemiology (CHARGE) (n=66). **B.** Comparison of current smoking DNA methylation effect sizes for CpGs with *P*<10^-4^ in lung adenocarcinoma and newborn blood from Pregnancy And Childhood Epigenetics (PACE) (n=24).


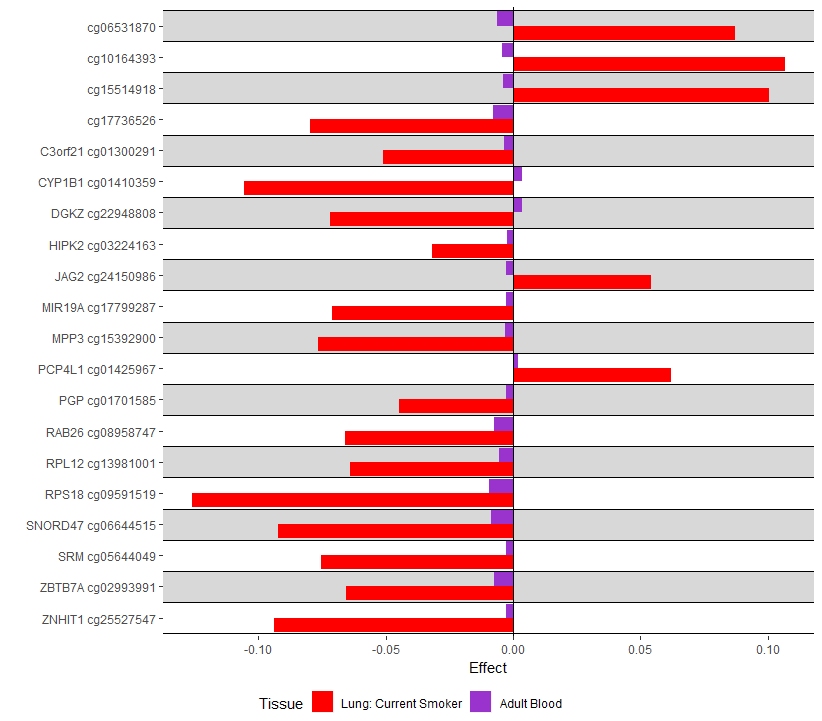


**Supplementary Figure 8.** Sensitivity analysis demonstrating DNA methylation is associated with smoking status (current versus never) in lung adenocarcinoma tissues from The Cancer Genome Atlas (TCGA) and in adult blood from the Cohorts for Heart and Aging Research in Genomic Epidemiology (CHARGE). There were 20 CpGs FDR<0.05 in adult blood, which did not meet the *P*<10^-4^ threshold in adult blood, but that did have *P*<10^-4^ in lung. Comparison of current smoking DNA methylation effect sizes for these CpGs across tissues (n=20).

| **A.**  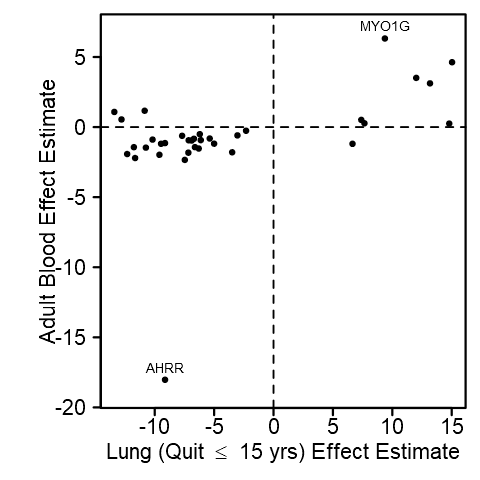 | **B.** 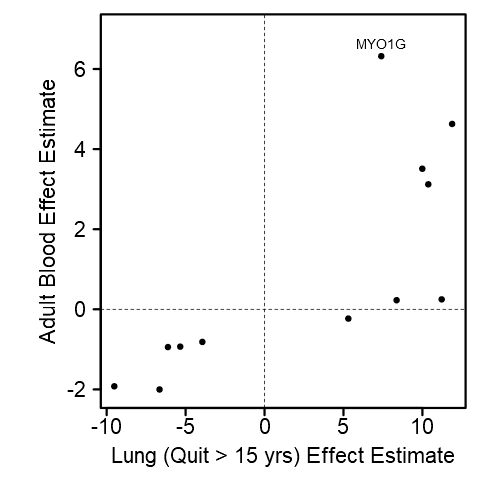 |
| --- | --- |
| **C.**  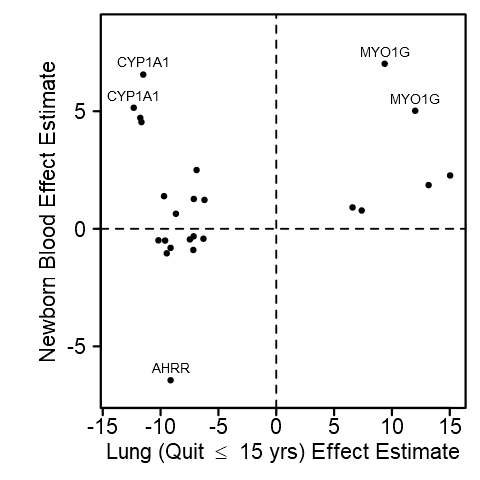 | **D.** 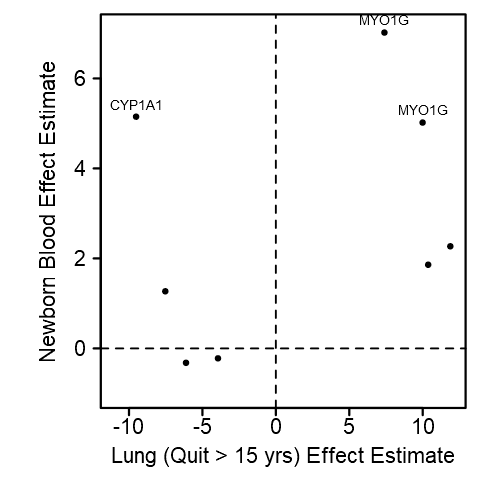 |

**Supplementary Figure 9.** Former smoking effect estimates in lung adenocarcinoma samples and blood for enriched sites. Adult blood (**A**, **B**). Newborn blood (**C**, **D**). Former smoking ≤ 15 years (**A**, **C**). Former smoking > 15 years (**B**, **D**).


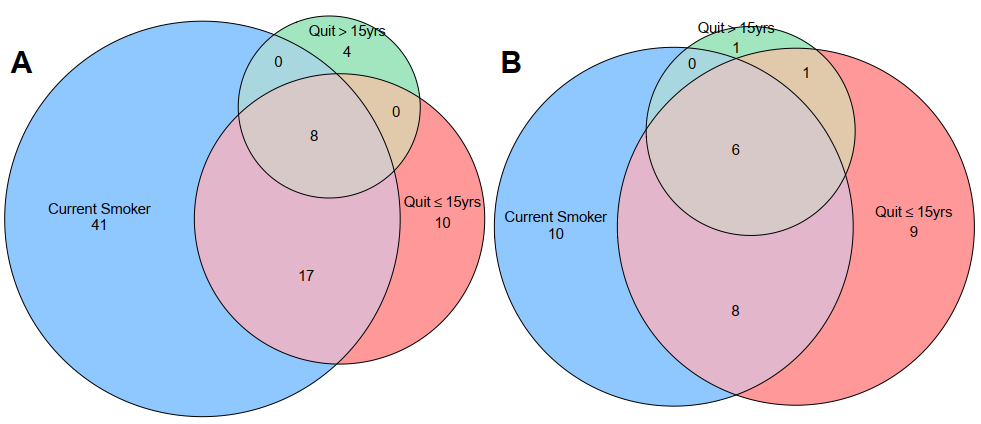


**Supplementary Figure 10.** **A**. DNA methylation sites associated with current smoking in adult blood that overlap with DNA methylation sites associated with smoking in lung adenocarcinoma, by lung sample exposure status. Relative to never smoking, current smoking (n=66 sites), recent former smoking (n=35), and longer former smoking (n=12). A total of n=8 sites had *P*<10^-4^ in adult blood and all categories of smoking in lung adenocarcinoma. **B**. DNA methylation sites associated with maternal smoking in newborn blood that overlap with DNA methylation sites associated with smoking in lung adenocarcinoma, by lung sample exposure status. Relative to never smoking, current smoking (n=24 sites), recent former smoking (n=24), and longer former smoking (n=8). A total of n=6 sites had *P*<10^-4^ in newborn blood and all categories of smoking in lung adenocarcinoma. Colors reflect smoke exposure level in lung adenocarcinoma (blue=current smokers; red=recently quit smoking; green=longer term former smokers).


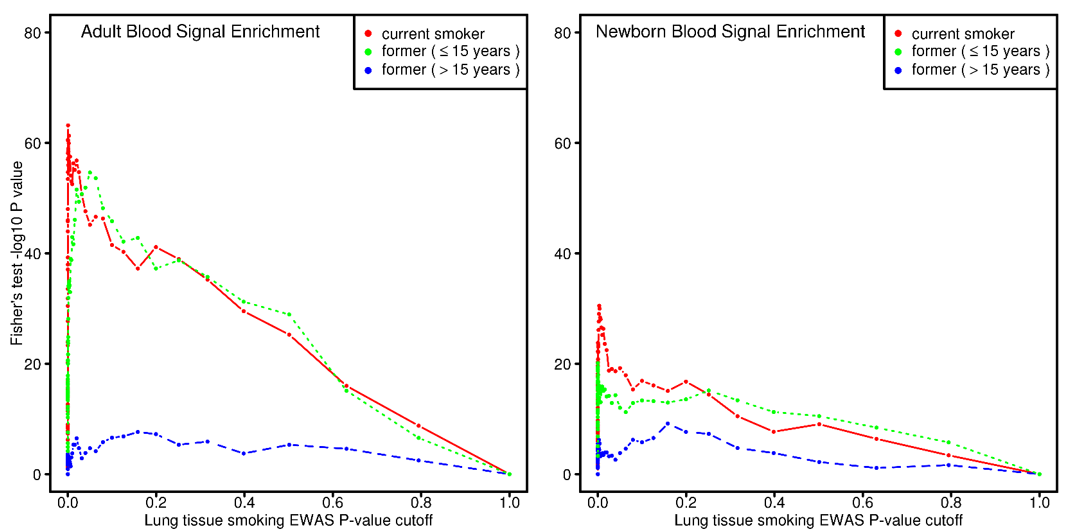


**Supplementary Figure 11.** Fisher’s enrichment test results comparing DNA methylation sites associated with level of smoking (current, former < 15 years, >15 years) versus never smoking in lung adenocarcinoma relative to blood (FDR<0.05). **A**. Adult blood DNA methylation smoking DNA methylation sites from the Cohorts for Heart and Aging Research in Genomic Epidemiology (CHARGE) consortium are enriched for lung adenocarcinoma DNA methylation smoking DNA methylation sites in The Cancer Genome Atlas. **B**. The smoking signature in newborn blood from the Pregnancy and Childhood Epigenetics (PACE) consortium is also enriched, but at a lower level compared to the adult signal. Results are plotted by current smoking (red), recent former smoking (green), and longer former smoking (blue) in lung adenocarcinoma.


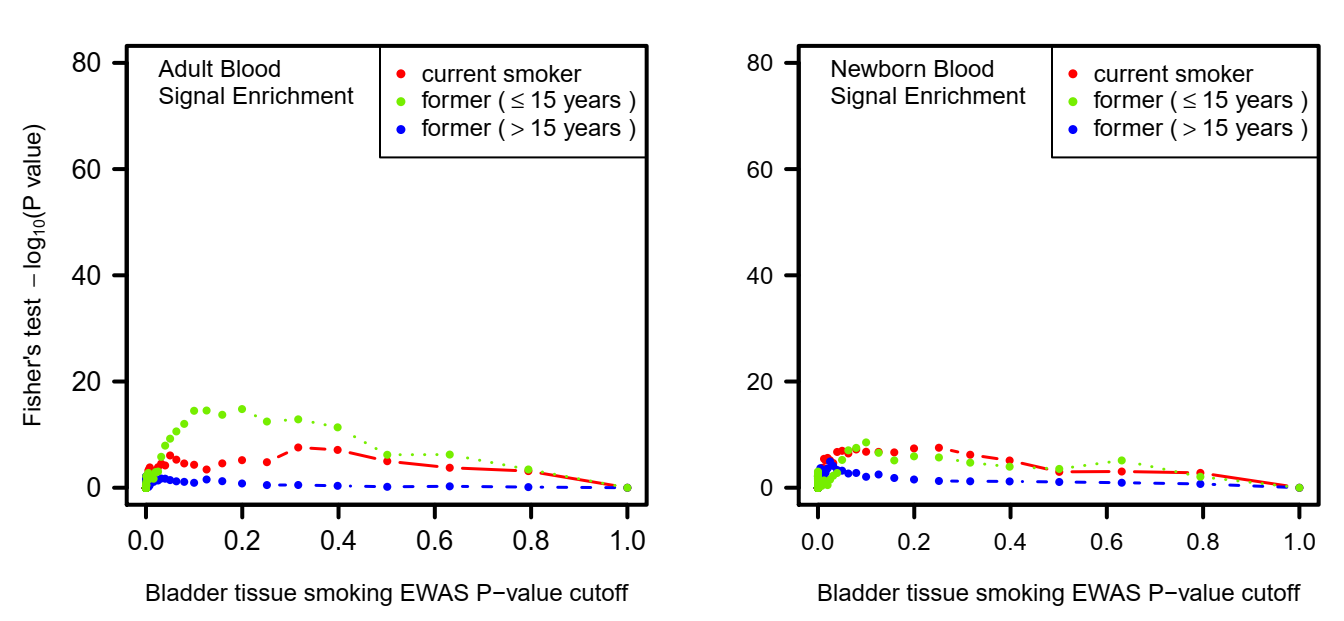


**Supplementary Figure 12.** Fisher’s enrichment test results comparing DNA methylation sites associated with level of smoking (current, former < 15 years, >15 years) versus never smoking in bladder carcinoma relative to blood (FDR<0.05). Results are plotted by current smoking (red), recent former smoking (green), and longer former smoking (blue) in bladder carcinoma. **A**. Adult blood DNA methylation smoking DNA methylation sites from the Cohorts for Heart and Aging Research in Genomic Epidemiology (CHARGE) consortium relative to lung adenocarcinoma DNA methylation smoking DNA methylation sites in The Cancer Genome Atlas. **B**. Newborn blood from the Pregnancy and Childhood Epigenetics (PACE) consortium relative to lung adenocarcinoma DNA methylation smoking DNA methylation sites in The Cancer Genome Atlas.

**Supplementary Table 1.** DNA methylation sites associated (*FDR* value < 0.05) with smoke exposure (versus never smokers) in The Cancer Genome Atlas lung adenocarcinoma tissue samples.

| **CpG** | **Chr** | **Position** | **Annotated Gene** | **Estimated Change in % Meth.** | **Std. Error** | **P-value** | **Average % Meth.** | **Gap Probe** |
| --- | --- | --- | --- | --- | --- | --- | --- | --- |
| **Current Smokers** | | | | | | | |  |
| cg12086028 | chr6 | 33241026 | VPS52;RPS18 | -15.12 | 2.37 | 5.53E-10 | 31.5 | N |
| cg27033919 | chr11 | 62622173 | SNORD30;SNORD29;SLC3A2;SLC3A2;SNORD31;SNORD28;SLC3A2;SLC3A2;SNHG1;SLC3A2 | -13.19 | 2.16 | 2.79E-09 | 13.4 | Y |
| cg18806997 | chr1 | 45242078 | SNORD46;RPS8;SNORD38A | -7.2 | 1.19 | 3.34E-09 | 9.6 | N |
| cg22132788 | chr7 | 45002486 | MYO1G | 13.31 | 2.24 | 7.16E-09 | 76.3 | N |
| cg16290996 | chr1 | 173835989 | GAS5;SNORD78;SNORD76;SNORD77;SNORD44;SNORD79 | -12.64 | 2.13 | 7.26E-09 | 21.8 | N |
| cg13985198 | chr1 | 45242073 | SNORD46;RPS8;SNORD38A | -8.22 | 1.39 | 7.63E-09 | 6 | Y |
| cg02607319 | chr7 | 45002112 |  | 12.95 | 2.23 | 1.36E-08 | 69.2 | N |
| cg07362537 | chr6 | 33240820 | VPS52;RPS18 | -13.18 | 2.35 | 3.96E-08 | 21.2 | Y |
| cg02905828 | chr11 | 62622234 | SNORD30;SNORD29;SLC3A2;SLC3A2;SNORD31;SNORD28;SLC3A2;SLC3A2;SNHG1;SLC3A2 | -13.25 | 2.37 | 4.36E-08 | 16.5 | N |
| cg11374355 | chr19 | 39925660 | RPS16 | -9.26 | 1.66 | 5.27E-08 | 42.1 | N |
| cg12973930 | chr19 | 57049695 | ZFP28 | -10.66 | 1.92 | 5.73E-08 | 11.5 | N |
| cg19089201 | chr7 | 45002287 | MYO1G | 11.88 | 2.16 | 7.42E-08 | 68 | N |
| cg09345320 | chr11 | 62622179 | SNORD30;SNORD29;SLC3A2;SLC3A2;SNORD31;SNORD28;SLC3A2;SLC3A2;SNHG1;SLC3A2 | -13.27 | 2.43 | 8.39E-08 | 13 | Y |
| cg05840553 | chr9 | 130212635 | RPL12;LRSAM1;LRSAM1 | -6.28 | 1.15 | 8.52E-08 | 6.8 | Y |
| cg24046474 | chr9 | 130212550 | RPL12;LRSAM1;LRSAM1 | -11.48 | 2.11 | 1.02E-07 | 20.5 | Y |
| cg04180046 | chr7 | 45002736 | MYO1G | 11.17 | 2.1 | 1.77E-07 | 57.8 | N |
| cg01410359 | chr2 | 38302230 | CYP1B1 | -10.55 | 1.99 | 1.94E-07 | 33.5 | N |
| cg05512483 | chr11 | 62621791 | SNORD30;SNORD22;SNORD29;SNORD31;SNHG1 | -11.64 | 2.2 | 2.04E-07 | 22 | N |
| cg05292954 | chr19 | 57050620 | ZFP28 | -12.07 | 2.3 | 2.67E-07 | 14.8 | N |
| cg17984022 | chr14 | 105156566 | INF2;INF2;INF2 | -5.04 | 0.97 | 3.59E-07 | 9.7 | Y |
| cg04783231 | chr19 | 57050834 | ZFP28 | -10.35 | 1.99 | 3.63E-07 | 25.8 | N |
| cg02932780 | chr6 | 31762353 | VARS | -9.19 | 1.78 | 3.94E-07 | 34.3 | N |
| cg12091542 | chr3 | 48700425 | CELSR3 | -9.22 | 1.8 | 4.79E-07 | 16.4 | N |
| cg11929643 | chr3 | 48700391 | CELSR3 | -12.95 | 2.55 | 6.17E-07 | 9.1 | N |
| cg01657995 | chr6 | 31804883 | C6orf48;C6orf48;SNORD52 | -9.99 | 1.97 | 6.32E-07 | 24.7 | N |
| cg12873476 | chr8 | 142402728 |  | -3.85 | 0.76 | 6.88E-07 | 35.7 | N |
| cg26283141 | chr6 | 33240471 | VPS52;RPS18 | -10.36 | 2.06 | 7.67E-07 | 37.1 | N |
| cg02725398 | chr3 | 9988144 | PRRT3 | -4.59 | 0.91 | 8.27E-07 | 17.6 | N |
| cg09736162 | chr3 | 48700443 | CELSR3 | -8.89 | 1.77 | 8.42E-07 | 9.1 | N |
| cg25157280 | chr3 | 48700498 | CELSR3 | -8.12 | 1.62 | 8.73E-07 | 5.9 | N |
| cg10573386 | chr3 | 48700375 | CELSR3 | -13.11 | 2.62 | 8.76E-07 | 9.1 | Y |
| cg09427809 | chr6 | 31804562 | C6orf48;C6orf48;SNORD52 | -9.08 | 1.82 | 9.08E-07 | 18.1 | Y |
| cg04388657 | chr9 | 127623377 | RPL35 | -9.72 | 1.95 | 1.01E-06 | 35.3 | N |
| cg06868511 | chr13 | 27504562 |  | -3.17 | 0.64 | 1.08E-06 | 14 | N |
| cg09799983 | chr2 | 38301756 | CYP1B1 | -22.95 | 4.65 | 1.27E-06 | 39.3 | N |
| cg02019774 | chr3 | 138658470 |  | 9.94 | 2.02 | 1.29E-06 | 30.6 | N |
| cg20408276 | chr2 | 38300586 | CYP1B1 | -16.26 | 3.32 | 1.48E-06 | 66.9 | N |
| cg11073255 | chr6 | 31762455 | VARS | -8.19 | 1.68 | 1.59E-06 | 13.6 | N |
| cg01851088 | chr3 | 48700337 | CELSR3;CELSR3 | -12.31 | 2.53 | 1.75E-06 | 8.4 | Y |
| cg11668917 | chr2 | 10586873 | SNORA80B;ODC1 | -9.44 | 1.94 | 1.82E-06 | 46.1 | N |
| cg06583813 | chr20 | 62710295 | RGS19;RGS19;OPRL1;OPRL1 | -6.89 | 1.42 | 1.84E-06 | 11.4 | N |
| cg17904852 | chr3 | 48700269 | CELSR3;CELSR3 | -10.92 | 2.25 | 1.85E-06 | 9.1 | N |
| cg02162897 | chr2 | 38300537 | CYP1B1 | -14.44 | 2.98 | 1.85E-06 | 75.1 | Y |
| cg03157040 | chr19 | 1861890 | KLF16 | -6.94 | 1.44 | 2.08E-06 | 9.9 | Y |
| cg01498999 | chrY | 16939383 | NLGN4Y;NLGN4Y;NLGN4Y | 5.37 | 1.11 | 2.15E-06 | 61.1 | N |
| cg13482209 | chr3 | 48700475 | CELSR3 | -12.31 | 2.57 | 2.45E-06 | 13 | N |
| cg10586870 | chr5 | 75722317 | IQGAP2 | -7.69 | 1.61 | 2.67E-06 | 34.5 | N |
| cg03045079 | chr14 | 39645334 | PNN | -3.43 | 0.73 | 3.72E-06 | 7.9 | N |
| cg06367459 | chr19 | 55898325 | RPL28;RPL28;RPL28;RPL28;RPL28 | -6.21 | 1.32 | 3.79E-06 | 12.8 | N |
| cg22040303 | chrX | 150912076 | CNGA2 | 6.13 | 1.31 | 3.85E-06 | 43.9 | N |
| cg02732508 | chr16 | 89628853 | RPL13;RPL13 | -11.61 | 2.48 | 3.96E-06 | 42.3 | N |
| cg26821115 | chr6 | 31804609 | C6orf48;C6orf48;SNORD52 | -6.76 | 1.44 | 4.04E-06 | 9.8 | N |
| cg04736217 | chr6 | 31804461 | C6orf48;C6orf48;SNORD52 | -10.93 | 2.35 | 4.62E-06 | 30.5 | N |
| cg19757631 | chr1 | 11118889 | SRM | -12.28 | 2.64 | 4.81E-06 | 60.4 | N |
| cg15075241 | chr2 | 10587052 | SNORA80B;ODC1 | -12.81 | 2.76 | 4.85E-06 | 59.8 | N |
| cg03607573 | chr11 | 93471889 | TAF1D | -9.69 | 2.09 | 4.86E-06 | 39.8 | N |
| cg26668608 | chr2 | 113931518 | PSD4;LOC440839 | 4.08 | 0.88 | 4.88E-06 | 55.3 | N |
| cg16355231 | chr1 | 2344979 | PEX10;PEX10 | -6.62 | 1.43 | 4.90E-06 | 18.5 | N |
| cg03043406 | chr1 | 45242356 | RPS8;SNORD38A | -10.9 | 2.35 | 4.98E-06 | 45.6 | N |
| cg18015301 | chr7 | 38624096 | AMPH;AMPH | 9.63 | 2.08 | 4.99E-06 | 74.5 | N |
| cg16052901 | chr6 | 33240864 | VPS52;RPS18 | -12.02 | 2.61 | 5.56E-06 | 42.4 | N |
| cg15484808 | chr6 | 33242542 | RPS18 | -11.1 | 2.41 | 5.84E-06 | 54.3 | N |
| cg19098932 | chr1 | 2345152 | PEX10;PEX10 | -8.07 | 1.76 | 6.02E-06 | 25.7 | Y |
| cg07849302 | chr10 | 114709887 | TCF7L2;TCF7L2;TCF7L2;TCF7L2;TCF7L2;TCF7L2 | -1.42 | 0.31 | 6.32E-06 | 8.4 | N |
| cg01103827 | chr21 | 40554221 | PSMG1;PSMG1 | -10.04 | 2.19 | 6.66E-06 | 35.9 | N |
| cg02993991 | chr19 | 4064177 | ZBTB7A | -6.58 | 1.44 | 6.93E-06 | 19.9 | Y |
| **Former Smokers (≤15 Years)** | | | | | | | |  |
| cg22132788 | chr7 | 45002486 | MYO1G | 15.03 | 2.05 | 1.57E-12 | 76.3 | N |
| cg02607319 | chr7 | 45002112 |  | 14.79 | 2.03 | 2.31E-12 | 69.2 | N |
| cg19089201 | chr7 | 45002287 | MYO1G | 13.17 | 1.97 | 9.73E-11 | 68 | N |
| cg04180046 | chr7 | 45002736 | MYO1G | 12.01 | 1.91 | 1.03E-09 | 57.8 | N |
| cg23160522 | chr15 | 75015787 | CYP1A1 | -8.68 | 1.44 | 4.34E-09 | 57.9 | N |
| cg18806997 | chr1 | 45242078 | SNORD46;RPS8;SNORD38A | -6.12 | 1.08 | 3.34E-08 | 9.6 | N |
| cg13985198 | chr1 | 45242073 | SNORD46;RPS8;SNORD38A | -7.14 | 1.27 | 3.69E-08 | 6 | Y |
| cg18092474 | chr15 | 75019302 | CYP1A1 | -12.32 | 2.21 | 4.65E-08 | 43.6 | N |
| cg01410359 | chr2 | 38302230 | CYP1B1 | -10.14 | 1.82 | 4.66E-08 | 33.5 | N |
| cg03224163 | chr7 | 139420300 | HIPK2;HIPK2 | -3.89 | 0.7 | 6.01E-08 | 83.1 | Y |
| cg09799983 | chr2 | 38301756 | CYP1B1 | -23.47 | 4.25 | 6.59E-08 | 39.3 | N |
| cg12803068 | chr7 | 45002919 | MYO1G | 9.37 | 1.72 | 9.84E-08 | 73.4 | N |
| cg00396865 | chr9 | 139620597 | SNHG7;SNORA43;SNHG7;SNHG7 | -7.69 | 1.45 | 2.06E-07 | 44.7 | N |
| cg12101586 | chr15 | 75019203 | CYP1A1 | -11.65 | 2.21 | 2.38E-07 | 30.7 | N |
| cg03595286 | chr9 | 110228216 |  | 9.13 | 1.79 | 5.17E-07 | 14.1 | N |
| cg09438320 | chr19 | 51053313 | LRRC4B | -6.4 | 1.27 | 8.16E-07 | 57.7 | N |
| cg12086028 | chr6 | 33241026 | VPS52;RPS18 | -10.75 | 2.17 | 1.08E-06 | 31.5 | N |
| cg16290996 | chr1 | 173835989 | GAS5;SNORD78;SNORD76;SNORD77;SNORD44;SNORD79 | -9.61 | 1.95 | 1.27E-06 | 21.8 | N |
| cg08733957 | chr1 | 24125298 | GALE;GALE;GALE | 7.59 | 1.57 | 1.91E-06 | 74.1 | N |
| **Former Smokers (>15 Years)** | | | | | | | |  |
| cg25157280 | chr3 | 48700498 | CELSR3 | -8.97 | 1.57 | 2.27E-08 | 5.9 | N |
| cg22132788 | chr7 | 45002486 | MYO1G | 11.88 | 2.17 | 8.43E-08 | 76.3 | N |
| cg09736162 | chr3 | 48700443 | CELSR3 | -9.35 | 1.71 | 9.34E-08 | 9.1 | N |
| cg02607319 | chr7 | 45002112 |  | 11.22 | 2.15 | 3.22E-07 | 69.2 | N |
| cg10936964 | chr4 | 13549687 | LOC285548 | -5.3 | 1.03 | 4.39E-07 | 14.3 | N |
| cg11929643 | chr3 | 48700391 | CELSR3 | -12.31 | 2.47 | 9.55E-07 | 9.1 | N |
| cg01851088 | chr3 | 48700337 | CELSR3;CELSR3 | -12.19 | 2.45 | 1.01E-06 | 8.4 | Y |
| cg13482209 | chr3 | 48700475 | CELSR3 | -12.36 | 2.49 | 1.02E-06 | 13 | N |
| cg12091542 | chr3 | 48700425 | CELSR3 | -8.63 | 1.74 | 1.07E-06 | 16.4 | N |
| cg19089201 | chr7 | 45002287 | MYO1G | 10.37 | 2.09 | 1.08E-06 | 68 | N |
| cg17904852 | chr3 | 48700269 | CELSR3;CELSR3 | -10.75 | 2.18 | 1.24E-06 | 9.1 | N |
| cg04180046 | chr7 | 45002736 | MYO1G | 9.99 | 2.03 | 1.27E-06 | 57.8 | N |
| cg20263853 | chr1 | 8824177 | RERE;RERE | 10.11 | 2.05 | 1.33E-06 | 61.6 | N |
| cg10573386 | chr3 | 48700375 | CELSR3 | -12.46 | 2.53 | 1.35E-06 | 9.1 | Y |

| **Supplementary Table 2.** Bivariate descriptive statistics in bladder carcinoma (N=372) from The Cancer Genome Atlas (TCGA) with DNA methylation data from the Illumina 450k array available. | | | | | |
| --- | --- | --- | --- | --- | --- |
|  | Never smokers | Current smokers | Former≤15 years | Former>15 years | P-value |
|  | N=108 | N=86 | N=69 | N=109 |  |
| Sex N(%) |  |  |  |  | 0.041 |
| Male | 69 (63.9%) | 67 (77.9%) | 51 (73.9%) | 87 (79.8%) |  |
| Age Mean(sd) | 68.0 (12.0) | 63.9 (9.58) | 66.1 (8.84) | 73.1 (8.48) | <0.001 |
| Smoking Pack Years Mean(sd) | - | 51.9 (88.4) | 39.3 (21.2) | 30.9 (26.9) | 0.056 |
| Missing (%) | - | 19 (22.1%) | 12 (17.4%) | 21 (19.3%) |  |
| Race N(%) |  |  |  |  | <0.001 |
| Unknown | 3 (2.8%) | 9 (10.5%) | 1 (1.45%) | 4 (3.67%) |  |
| Asian | 24 (22.2%) | 13 (15.1%) | 5 (7.25%) | 1 (0.92%) |  |
| Black | 4 (3.7%) | 5 (5.81%) | 6 (8.70%) | 5 (4.59%) |  |
| White | 77 (71.3%) | 59 (68.6%) | 57 (82.6%) | 99 (90.8%) |  |
| Cancer stage N(%) |  |  |  |  | 0.07 |
| I | 0 (0.00%) | 1 (1.16%) | 0 (0.00%) | 1 (0.92%) |  |
| II | 40 (37.0%) | 29 (33.7%) | 25 (36.2%) | 25 (22.9%) |  |
| III | 38 (35.2%) | 35 (40.7%) | 20 (29.0%) | 36 (33.0%) |  |
| IV | 30 (27.8%) | 21 (24.4%) | 24 (34.8%) | 47 (43.1%) |  |

**Supplementary Table 3.** Pathways with *P*<10^-3^ for genes implicated through DNA methylation of current smokers versus never smokers only in lung adenocarcinoma (CpG sites with *P*<10^-4^) and not in adult or newborn blood.

| **GO Term ID** | **Pathway** | **Genes in Pathway** | **Number of Genes Targeted by CpGs** | **P-value** | **FDR** | **Genes Targeted by CpGs** |
| --- | --- | --- | --- | --- | --- | --- |
| GO:0010613 | positive regulation of cardiac muscle hypertrophy | 37 | 4 | 3.98E-05 | 0.295311 | MIR17, MIR19A, MIR19B1, MIR20A |
| GO:0010665 | regulation of cardiac muscle cell apoptotic process | 46 | 4 | 9.48E-05 | 0.301042 | MIR17, MIR19A, MIR19B1, MIR20A |
| GO:0000956 | nuclear-transcribed mRNA catabolic process | 207 | 6 | 0.000744 | 0.828461 | PAN3, RPS16, RPS23, RPS27A, SKIV2L, RPL14 |
| GO:0006614 | SRP-dependent cotranslational protein targeting to membrane | 91 | 4 | 0.001299 | 0.997279 | RPS16, RPS23, RPS27A, RPL14 |
| GO:0019083 | viral transcription | 177 | 5 | 0.002319 | 0.997279 | RPS16, RPS23, RPS27A, NELFE, RPL14 |
| GO:0046184 | aldehyde biosynthetic process | 15 | 2 | 0.002685 | 0.997279 | GPI, CACNA1H |
| GO:0035278 | miRNA mediated inhibition of translation | 56 | 3 | 0.003091 | 0.997279 | MIR17, MIR19B1, MIR20A |
| GO:0006412 | translation | 818 | 11 | 0.00349 | 0.997279 | IGF2BP2, MIR17, MIR18A, MIR19A, MIR19B1, MIR20A, RPS16, RPS23, RPS27A, RPL14, ZNF598 |
| GO:0002347 | response to tumor cell | 20 | 2 | 0.004777 | 0.997279 | IGFBPL1, NECTIN2 |
| GO:1904707 | positive regulation of vascular smooth muscle cell proliferation | 23 | 2 | 0.006297 | 1 | MIR17, MIR20A |
| GO:0006297 | nucleotide-excision repair, DNA gap filling | 23 | 2 | 0.006297 | 1 | POLD1, RPS27A |
| GO:0006979 | response to oxidative stress | 422 | 7 | 0.00649 | 1 | CAMKK2, DGKK, GUCY1B3, MIR17, MIR19A, BTK, WRN |
| GO:0000731 | DNA synthesis involved in DNA repair | 74 | 3 | 0.006758 | 1 | POLD1, RPS27A, WRN |

**Supplementary Table 4.** Pathways with *P<*10^-3^ for genes implicated through DNA methylation of current smokers versus never smokers in lung adenocarcinoma and in blood meta-analysis (CpG sites with *P*<10^-4^).

| GO Term ID | Pathway | Genes in Pathway | Number of Genes Targeted by CpGs | P-value | FDR | Genes Targeted by CpGs |
| --- | --- | --- | --- | --- | --- | --- |
| GO:0006614 | SRP-dependent cotranslational protein targeting to membrane | 91 | 9 | 1.59E-12 | 2.81E-08 | RPL35, RPL3, RPL13, RPL23A, RPL28, RPLP2, RPS8, RPS18, RPL23 |
| GO:0000184 | nuclear-transcribed mRNA catabolic process, nonsense-mediated decay | 119 | 9 | 1.86E-11 | 6.90E-08 | RPL35, RPL3, RPL13, RPL23A, RPL28, RPLP2, RPS8, RPS18, RPL23 |
| GO:0019083 | viral transcription | 177 | 9 | 6.57E-10 | 1.33E-06 | RPL35, RPL3, RPL13, RPL23A, RPL28, RPLP2, RPS8, RPS18, RPL23 |
| GO:0006413 | translational initiation | 183 | 9 | 8.82E-10 | 1.51E-06 | RPL35, RPL3, RPL13, RPL23A, RPL28, RPLP2, RPS8, RPS18, RPL23 |
| GO:0006364 | rRNA processing | 255 | 9 | 1.61E-08 | 1.92E-05 | RPL35, RPL3, RPL13, RPL23A, RPL28, RPLP2, RPS8, RPS18, RPL23 |
| GO:0034660 | ncRNA metabolic process | 568 | 9 | 1.28E-05 | 0.008407 | RPL35, RPL3, RPL13, RPL23A, RPL28, RPLP2, RPS8, RPS18, RPL23 |
| GO:0016071 | mRNA metabolic process | 790 | 9 | 0.000165 | 0.087593 | RPL35, RPL3, RPL13, RPL23A, RPL28, RPLP2, RPS8, RPS18, RPL23 |
| GO:0010629 | negative regulation of gene expression | 1882 | 14 | 0.000207 | 0.104993 | RPL35, ZBTB7A, AHRR, RPL3, RPL13, RPL23A, RPL28, RPLP2, RPS8, RPS18, NSD1, TCF7L2, EHMT1, RPL23 |
| GO:0006396 | RNA processing | 917 | 9 | 0.000496 | 0.218417 | RPL35, RPL3, RPL13, RPL23A, RPL28, RPLP2, RPS8, RPS18, RPL23 |
| GO:0043603 | cellular amide metabolic process | 1146 | 10 | 0.000589 | 0.252404 | RPL35, RPL3, RPL13, RPL23A, RPL28, RPLP2, RPS8, RPS18, XPNPEP1, RPL23 |

**Supplementary Table 5.** Genes with DNA methylation sites associated with current smoke exposure versus never smokers, annotated to common genes across lung adenocarcinoma tissue (*P*<10^-4^), adult blood (*P*<10^-4^), and newborn blood (*P*<10^-4^). The 14 CpGs reaching genome wide significance (*P*<10^-7^) in lung adenocarcinoma are bold.

| Gene | CpG in Lung | CpG in Adult Blood | CpG in Newborn Blood | Number of Probes on Array Annotated to Gene |
| --- | --- | --- | --- | --- |
| *AHRR* | cg05575921, cg04135110, cg16896326 | cg00976097, cg01970407, cg02385153, cg03604011, cg05575921, cg06802630, cg08858540, cg09084391, cg10841124, cg14453201, cg14454127, cg14647125, cg14714797, cg15179499, cg16294152, cg17287155, cg22103736, cg22698028, cg23067299, cg25004427, cg26076054, cg26529655, cg26850624, cg00300637, cg00401753, cg01097768, cg01899089, cg03991871, cg04135110, cg04141806, cg04551776, cg08491376, cg08556107, cg08606254, cg09338136, cg11554391, cg11557553, cg11902777, cg12202185, cg12207033, cg12251573, cg12806681, cg14448919, cg14807090, cg14817490, cg16049691, cg16219322, cg16336872, cg16896326, cg17924476, cg18541609, cg19039843, cg21161138, cg22356527, cg22937882, cg23576855, cg23916896, cg24064903, cg24090911, cg24688690, cg24891125, cg24980413, cg25648203, cg26703534, cg26954197 | cg00976097, cg01970407, cg05575921, cg09078081, cg10841124, cg17401179, cg22698028, cg23067299, cg26529655, cg26850624, cg01097768, cg01899089, cg02356223, cg03991871, cg04141806, cg04551776, cg07448928, cg08606254, cg11554391, cg11557553, cg11902777, cg12202185, cg12207033, cg12806681, cg14817490, cg16172278, cg17924476, cg21161138, cg22937882, cg23916896, cg24090911, cg24688690, cg24980413, cg25648203, cg26703534, cg26954197 | 138 |
| *ATP5I* | cg20576162 | cg23970089, cg26210267 | cg26210267 | 13 |
| *C6orf48* | cg09427809, cg01657995, cg04736217, cg05789250, cg07089321, cg26821115 | cg06482498, cg04736217, cg05789250, cg13541527, cg20109054 | cg11931646, cg13541527 | 51 |
| *CAMTA1* | cg03580787 | cg10626316, cg21144493, cg04218548, cg05129050, cg05285441, cg06830167, cg09889997, cg10331119, cg23972860, cg27557829 | cg06077003, cg24999973, cg00452133, cg11755201, cg12097989, cg20800117 | 249 |
| *CMSS1* | cg21589858 | cg13612275, cg15554421, cg27384355 | cg04411201 | 72 |
| *CYP1A1* | cg12101586, cg18092474, cg23160522 | cg00213123, cg12101586, cg13570656, cg20004910, cg22549041, cg11924019, cg18092474, cg23680900 | cg00213123, cg12101586, cg13570656, cg22549041, cg26516004, cg05549655, cg11924019, cg17852385, cg18092474, cg23160522, cg23680900, cg23727072 | 34 |
| *EHMT1* | cg09879186 | cg05616472 | cg05616472 | 69 |
| *FILIP1L* | cg21589858 | cg13612275, cg15554421 | cg04411201 | 50 |
| *GAS5* | cg03044573, cg06644515, cg07177756, **cg16290996**, cg17025683 | cg03044573, cg06644515, cg07177756, cg16290996, cg17025683 | cg16290996, cg17025683 | 29 |
| *INF2* | cg17984022 | cg01836137, cg02305961, cg11730703, cg13578465, cg18425377, cg13590876, cg22090592, cg23998240, cg27286011 | cg13590876 | 57 |
| *JAG2* | cg24150986 | cg15421321, cg24150986 | cg05335315 | 48 |
| *KIF26B* | cg14792075 | cg05034124, cg12720965, cg15358723, cg24490133, cg25683662 | cg09368188 | 139 |
| *MIR548G* | cg21589858 | cg13612275 | cg04411201, cg24198004, cg24757159 | 68 |
| *MYO1G* | **cg19089201**, **cg22132788**, cg04180046, cg12803068 | cg19089201, cg22132788, cg04180046, cg07826859, cg09948419, cg12803068 | cg19089201, cg22132788, cg04180046, cg12803068 | 12 |
| *NSD1* | cg08947551 | cg17493885, cg18121224, cg19731612, cg24711224 | cg17493885, cg18121224 | 23 |
| *PAG1* | cg01771871 | cg02520639, cg09973676, cg19004971 | cg01771871, cg05525416 | 25 |
| *RASSF5* | cg12691994 | cg17220933, cg01442843, cg02035751, cg04144533, cg06872036, cg06949439, cg07380021, cg14163311 | cg01442843, cg02035751, cg06949439, cg19638572 | 42 |
| *RPL13* | cg02732508 | cg01035068, cg01995548, cg02732508 | cg01995548, cg02732508 | 14 |
| *RPL23* | cg05780139 | cg05174975, cg05780139, cg25615944 | cg05174975 | 15 |
| *RPL23A* | cg16565409, cg02650512, cg08165291, cg15391464 | cg16565409, cg00639656, cg14173815, cg15036326, cg20927567 | cg16565409, cg02650512 | 28 |
| *RPL28* | cg06367459 | cg06367459, cg08233235 | cg06367459 | 17 |
| *RPL3* | cg21649604 | cg01882930, cg04982781, cg09282085, cg15306060, cg21649604, cg24542766, cg25386611 | cg21649604, cg23141902 | 33 |
| *RPL35* | cg04388657 | cg04388657, cg04761231 | cg04388657 | 6 |
| *RPLP2* | cg01485797 | cg19165216, cg00330825, cg19216994 | cg01485797 | 24 |
| *RPS18* | cg09591519, **cg07362537**, cg11222065, **cg12086028**, cg15484808, cg16052901, cg25636481, cg26283141, cg27182159 | cg00694180, cg09591519, cg03702686, cg07362537, cg07556599, cg08014182, cg08090835, cg10399946, cg11222065, cg11682350, cg12086028, cg12583553, cg14266999, cg15484808, cg16052901, cg21166347, cg22322679, cg25636481, cg26283141, cg27182159 | cg21096118, cg07362537, cg11222065, cg11682350, cg12086028, cg14266999, cg16052901, cg26283141, cg27182159 | 63 |
| *RPS8* | **cg13985198**, cg03043406, **cg18806997** | cg13985198, cg03043406, cg17755386, cg18806997, cg22433141 | cg13985198, cg01881870, cg03043406, cg17755386 | 21 |
| *RXRB* | cg17567838 | cg00031105, cg01536956, cg03963919, cg04772644, cg14362925, cg16114213, cg17567838, cg24470206, cg25535027 | cg16425713 | 90 |
| *SFXN5* | cg23396786 | cg10040131 | cg10040131 | 38 |
| *SNHG1* | **cg02905828**, cg05512483, cg07297802, **cg09345320**, **cg27033919** | cg03431111, cg05512483, cg09448652, cg15172739, cg15345437, cg18159646 | cg09448652, cg15172739 | 31 |
| *SNHG15* | cg16459265 | cg00953154, cg03440944, cg16459265 | cg16459265 | 13 |
| *SNORA52* | cg01485797 | cg19165216, cg00330825, cg19216994 | cg01485797 | 8 |
| *SNORA9* | cg16459265 | cg00953154, cg16459265 | cg16459265 | 11 |
| *SNORD139* | cg21649604 | cg01882930, cg04982781, cg09282085, cg15306060, cg21649604, cg24542766 | cg21649604 | 8 |
| *SNORD22* | cg05512483 | cg03431111, cg05512483, cg09448652, cg15172739, cg15345437, cg18159646 | cg09448652, cg15172739 | 6 |
| *SNORD30* | **cg02905828**, cg05512483, cg07297802, **cg09345320**, **cg27033919** | cg03431111, cg05512483, cg09448652, cg15172739, cg18159646 | cg09448652, cg15172739 | 9 |
| *SNORD31* | **cg02905828**, cg05512483, **cg09345320**, **cg27033919** | cg03431111, cg05512483, cg09448652, cg15172739, cg15345437, cg18159646 | cg09448652, cg15172739 | 9 |
| *SNORD38A* | **cg13985198**, cg03043406, **cg18806997** | cg13985198, cg03043406, cg17755386, cg18806997, cg22433141 | cg13985198, cg03043406, cg17755386 | 5 |
| *SNORD42A* | cg02650512 | cg00639656, cg14173815, cg20927567 | cg02650512 | 7 |
| *SNORD44* | cg03044573, cg07177756, **cg16290996**, cg17025683 | cg03044573, cg07177756, cg16290996, cg17025683 | cg16290996, cg17025683 | 6 |
| *SNORD46* | **cg13985198**, **cg18806997** | cg13985198, cg17755386, cg18806997 | cg13985198, cg01881870, cg17755386 | 15 |
| *SNORD4A* | cg16565409, cg02650512 | cg16565409, cg14173815, cg15036326, cg20927567 | cg16565409, cg02650512 | 6 |
| *SNORD52* | cg09427809, cg01657995, cg04736217, cg05789250, cg07089321, cg26821115 | cg04736217, cg05789250, cg13541527, cg20109054 | cg11931646, cg13541527 | 11 |
| *SNORD76* | cg07177756, **cg16290996**, cg17025683 | cg07177756, cg16290996, cg17025683 | cg16290996, cg17025683 | 18 |
| *SNORD77* | cg07177756, **cg16290996**, cg17025683 | cg07177756, cg16290996, cg17025683 | cg16290996, cg17025683 | 10 |
| *SNORD78* | cg03044573, cg06644515, cg07177756, **cg16290996**, cg17025683 | cg03044573, cg06644515, cg07177756, cg16290996, cg17025683 | cg16290996, cg17025683 | 6 |
| *SNORD79* | cg03044573, cg06644515, **cg16290996** | cg03044573, cg06644515, cg16290996 | cg16290996 | 4 |
| *TCF7L2* | cg07849302, cg14562081 | cg00159523, cg03339956, cg04322596, cg07168930, cg11748187, cg15624624, cg18464364, cg27162705 | cg00159523, cg07168930, cg18842107 | 80 |
| *TRIM2* | cg20818457, cg25837904 | cg02832697, cg05973772, cg12793610, cg23820816 | cg12793610, cg16708623 | 71 |
| *VPS52* | **cg07362537**, cg11222065, **cg12086028**, cg16052901, cg26283141 | cg07362537, cg11222065, cg11682350, cg12086028, cg14266999, cg16052901, cg17334970, cg20595453, cg21166347, cg26283141, cg26987613 | cg21096118, cg07362537, cg11222065, cg11682350, cg12086028, cg14266999, cg16052901, cg26283141 | 135 |
| *XPNPEP1* | cg03088705 | cg04350202, cg09352789 | cg09352789 | 20 |
| *XXYLT1* | cg01300291 | cg02504211, cg15251319, cg01300291, cg02650080, cg06706813 | cg06041567, cg15722952 | 86 |
| *ZBTB7A* | cg02993991, cg07328519 | cg02993991, cg13705556, cg02218768 | cg02218768 | 43 |
